# Following Pediatric and Adult IBD Patients through the COVID-19 Pandemic: Changes in Psychosocial Burden and Perception of Infection Risk and Harm over Time

**DOI:** 10.3390/jcm10184124

**Published:** 2021-09-13

**Authors:** Leandra Koletzko, Elisabeth Klucker, Thu Giang Le Thi, Simone Breiteneicher, Raquel Rubio-Acero, Lukas Neuhaus, Reneé G. Stark, Marie Standl, Andreas Wieser, Helga Török, Sibylle Koletzko, Tobias Schwerd

**Affiliations:** 1 Department of Medicine II, LMU Klinikum, 81377 Munich, Germany; leandra.koletzko@med.uni-muenchen.de (L.K.); Simone.Breiteneicher@med.uni-muenchen.de (S.B.); Lukas.neuhaus@med.uni-muenchen.de (L.N.); helga.toeroek@med.uni-muenchen.de (H.T.); 2 Department of Pediatrics, Dr von Hauner Kinderspital, LMU Klinikum, 80337 Munich, Germany; elisabeth.klucker@med.uni-muenchen.de (E.K.); Thu_Giang.Le_Thi@med.uni-muenchen.de (T.G.L.T.); tschwerd@med.lmu.de (T.S.); 3 Division of Infectious Diseases and Tropical Medicine, LMU Klinikum, 80802 Munich, Germany; Raquel.Rubio@med.uni-muenchen.de (R.R.-A.); wieser@mvp.lmu.de (A.W.); 4 Institute of Health Economics and Healthcare Management, Helmholtz Zentrum München, 85764 Neuherberg, Germany; r.stark@helmholtz-muenchen.de; 5 Institute of Epidemiology, Helmholtz Zentrum München, German Research Center for Environmental Health, 85764 Neuherberg, Germany; marie.standl@helmholtz-muenchen.de; 6 Department of Pediatrics, Gastroenterology and Nutrition, School of Medicine Collegium Medicum University of Warmia and Mazury, 10-719 Olsztyn, Poland

**Keywords:** Crohn’s disease, ulcerative colitis, psychosocial stress, COVID-19, SARS-CoV-2

## Abstract

Background: COVID-19-associated restrictions impact societies. We investigated the impact in a large cohort of inflammatory bowel disease (IBD) patients. Methods: Pediatric (pIBD) and adult patients and pIBD parents completed validated questionnaires for self-perceived stress (Perceived Stress Questionnaire, PSQ) and quality of life from July to October 2020 (1st survey) and March to April 2021 (2nd survey). Analyses were stratified by age groups (6–20, >20–40, >40–60, >60 years). Perceived risk of infection and harm from COVID-19 were rated on a 1–7 scale. An index for severe outcome (SIRSCO) was calculated. Multivariable logistic regression analysis was performed. Results: Of 820 invited patients, 504 (62%, 6–85 years) patients and 86 pIBD parents completed the 1st, thereof 403 (80.4%) the 2nd survey. COVID-19 restrictions resulted in cancelled doctoral appointments (26.7%), decreased physical activity, increased food intake, unintended weight gain and sleep disturbance. PSQ increased with disease activity. Elderly males rated lower compared to females or younger adults. PSQ in pIBD mothers were comparable to moderate/severe IBD adults. Infection risk and harm were perceived high in 36% and 75.4%. Multivariable logistic models revealed associations of higher perceived risk with >3 household members, job conditions and female gender, and of perceived harm with higher SIRSCO, unintended weight change, but not with gender or age. Cancelled clinic-visits were associated with both. SARS-CoV-2 antibodies prior 2nd infection wave were positive in 2/472 (0.4%). Conclusions: IBD patients report a high degree of stress and self-perceived risk of complications from COVID-19 with major differences related to gender and age. Low seroprevalence may indicate altered immune response.

## 1. Introduction

Worldwide restrictions in social contacts and mobility have been introduced to prevent the spread of the potentially deadly coronavirus disease (COVID-19) and to protect vulnerable population parts such as elderly people and those with certain underlying conditions. In Germany, the first confirmed COVID-19-cases were diagnosed at the end of January 2020 in Munich [1]. Although the transmission chain of this COVID cluster was successfully interrupted, a wave of SARS-CoV2-infections spread in March 2020 accelerated by many infected individuals returning from skiing holidays in Austria and Northern Italy, which had high infection rates. On March 16, Bavaria as the first state in Germany, announced a strict lockdown including closure of schools and daycares. All hospitals were ordered to restrict elective outpatient visits, elective endoscopic and surgical procedures, and devote all resources to emergencies and COVID-19 patients. This induced a high degree of uncertainty and anxiety in patients with inflammatory bowel disease (IBD) since most of them require long-term immunosuppressive therapy. During the first infection wave of the pandemic, information on the impact of IBD-medication on risk to acquire the infection, on the severity of disease course and on the mortality from COVID-19 was missing. Over time, data became available from published case series [2], observational studies [3], international registries [4,5,6] and finally a meta-analysis [7] that allowed risk estimation for certain drugs and co-morbidities, and the development of recommendations for IBD patients and their health care providers [6,8].

In Munich, a large prospective population-based cohort study (KoCo19), funded by the Bavarian ministry of science and culture, was performed to capture the burden of the pandemic. KoCo19 includes almost 3000 representative households with 5313 participants and surveyed socioeconomic factors, comorbidities and seroprevalence for SARS-CoV-2 antibodies [9,10]. In parallel, we recruited pediatric (pIBD) and adult IBD patients and pIBD parents to an IBD cohort across the age continuum (KoCo19-IBD) and assessed similar factors as KoCo19 as well as IBD and health care related factors. We used validated tools for IBD-related quality of life and psychosocial stress. We aimed to better understand the burden of the pandemic on IBD-related health care, psychosocial consequences, changes in diet and physical activity, work and school life, and self-perceived risk and harm related to COVID-19. We also assessed seroconversion rate prior the start of the second infection wave in October 2020.

## 2. Materials and Methods

### 2.1. Participants

After searching the electronic health record system of our tertiary IBD center, we invited all patients (*N* = 869) seen between June 2018 and June 2020 with a diagnosis of Crohn’s disease (CD), ulcerative colitis (UC), or IBD-unclassified (IBD-u) to participate in our prospective survey (Figure S1). Study information and consent forms were sent by surface mail mid-July to identified IBD patients and their caregivers, if younger than 18 years of age, with additional age-appropriate consent forms. Each patient received a personalized QR code (two dimensional code) to access the online version of the questionnaires, paper versions were provided on request. All responders were invited mid-February 2021 to a follow up survey to identify changes over time. The 1st survey covered the time from start of the pandemic, defined as 1 March 2020, until end of summer, the 2nd survey covered the beginning of the 2nd infection wave, defined as 1 November 2020 reaching into the 3rd wave until data cut in April 2021. Separate consents could be provided for the questionnaires and for serological testing for SARS-CoV-2 antibody concentrations.

The ethical committee of the Faculty of Medicine at LMU Munich approved the study protocol including data protection concept on 9 July 2020 (IRB approval No. 20–523) and the follow up survey on 12 February 2021. Written informed consent was obtained from all participating IBD patients aged 7 years and older, and both parents in pediatric patients <18 years of age.

### 2.2. Questionnaires

Different sets of questionnaires were provided to adult IBD patients, to parents of affected children aged <9 years and to those with children aged 9 to 17 years. Whenever possible, questions were based on pre-existing validated questionnaire instruments. Most epidemiological and socioeconomic questions were identical to those used in the population-based Munich KoCo19 study [9]. The survey included the following domains:

- Baseline characteristics including age, sex, country of birth, height and weight, area of living (urban or rural);
- Vaccination status for vaccinations against measles, mumps, rubella, varicella, zoster, pneumococcus and COVID-19 (2nd survey only). At both time points we also asked for influenza vaccinations in the last winter season (prior and during pandemic) and whether participants and healthy household members would choose to be vaccinated against COVID-19, if offered;
- Socio-economic status relating to the living situation, size of flat/house and room number, number and age range of household members, employment status, educational level and household income [9];
- IBD-related questions including IBD phenotype, age at diagnosis, complications, current disease activity and disease flares during the pandemic and detailed information on medication and non-drug supportive therapy for IBD and non-IBD related comorbidities;
- COVID-19 related questions regarding symptoms suggestive for COVID-19 within the last two weeks and since the beginning of the pandemic as well as all previous PCR tests from nasopharyngeal swabs and serology for SARS-CoV-2 antibodies in patients and household members [9]. Furthermore, this section included questions on employment situation, jobs with high number of in-person contacts (teacher, nursing home, health care professionals, grocery, pharmacy, police etc.) and therefore with potentially higher risk of COVID-19 infection, and any conditions or comorbidity with known increased risk for severe COVID-19 outcome;
- Impact on health care utilization for IBD, changes in medication, missed or cancelled clinic visits and diagnostic procedures due to the pandemic and experience with telemedicine;
- Effects on daily life relating to the situation at home, at work or at school, in the family, use of public transport, physical activity, sleeping and eating behavior, alcohol and tobacco habits, involuntary or voluntary weight change, general well-being and loneliness;
- Effects on psychological stress and quality of life were assessed with validated instruments. We used the German version of the Perceived Stress Questionnaire (PSQ) [11,12], which assesses subjectively experienced stress during the last 4 weeks in four domains (“worries”, “tension”, “joy”, demands”) with five items each and a total score ranging from 0 to 100. The domain “joy”, which is positively coded, is transformed into “lack of joy” to calculate the total PSQ score. A high PSQ score reflects a high level of perceived stress. Adult IBD patients and both pIBD parents reflecting their own situation answered the PSQ. To assess IBD-related quality of life, the Short Inflammatory Bowel Disease Questionnaire (SIBDQ) was used for adults [13,14] and the IMPACT III for pediatric patients [15,16]. Results are shown as mean with 95% CI for PSQ, and as mean with SD for SIBDQ and IMPACT III, in agreement with published references [13,15,17].
- Patients’ self-perceived risk and harm of COVID-19: On a scale from 1 (low) to 7 (high), adult patients estimated their risk to acquire COVID-19 infection (perceived risk) and their risk for a severe disease course if they get infected (perceived harm). Parents judged perceived risk and harm for their IBD affected child. Values of <3 and >3 were considered low and high perceived risk/harm, respectively.

License to use validated questionnaires: Use of the Inflammatory Bowel Disease Questionnaire, authored by Dr. Jan Irvine et al., was made under license from McMaster University, Hamilton, Canada. The use of the German version of the IMPACT III and PSQ Questionnaires was indicated in written agreement to the respective authors.

### 2.3. Scoring for Increased Risk for Severe COVID-19 Outcomes (SIRSCO)

We developed a Scoring index for Increased Risk for Severe COVID-19 Outcome (SIRSCO) to assess published risk factors associated with severe COVID-19 outcomes (hospitalization and death) such as age, co-morbidities and medications (Figure S2) [18]. Although older age is highly associated with severe COVID-19 outcomes, this is biased by the increasing risks for age related co-morbidities [19]. Our SIRSCO index assigned one point for each of the following: age over 70 years, treatment with any immunosuppressive drug [6,20,21], and for each co-morbidity including obesity defined as body mass index (BMI) >30 kg/m^2^ [22,23,24], diabetes [22,25], chronic liver, lung or heart disease [22], stroke [26], hypertension [7,27], renal insufficiency [25,28] and cancer [6,21,22,25,29]. Post-transplantation status was assigned 2 points [25,30]. Participants were divided into five SIRSCO categories: 0 points: no risk, 1 point: mild, 2 points: moderate, 3–4 points: severe and >5 very severe risk (Figure S2).

### 2.4. SARS-CoV2 Serology

Serum samples collected from August to mid-October 2020 were tested for IgG antibodies to SARS-CoV-2 (Elecsys Anti-SARS-CoV-2 Roche anti-N pan-Ig, Roche, Mannheim, Germany) in the same lab as in the KoCo19-study [10]. Seropositivity was defined according to the manufacturer ≥1.00 (reactive).

### 2.5. Statistical Analysis

Descriptive statistics were used to describe demographical and clinical characteristics of the total cohort stratified into four age groups: 6–20, >20–40, >40–60 and >60 years of age. Continuous variables were reported as mean (95% CI) or mean (SD) for normally distributed variables and median (interquartile range from 25th to 75th quartile, IQR) for non-normally distributed variables, while categorical variables are presented as frequency and proportion in percent (%). A continuous variable is considered as normally distributed, if the *p*-value of Shapiro–Wilk test is greater than 0.05 and if normal quantile-quantile plots show no serious deviation of the data points from the fitted line. To determine statistically significant differences between groups, we performed Mann–Whitney-*U*-test for continuous variables, while Pearson’s Chi-square test, or Fisher’s exact test for categorical variables. To compare the PSQ score of the mother vs. PSQ score of the father of the same pediatric patient, we used Wilcoxon signed-rank test or paired Student’s t-test where applicable.

To investigate significant changes in patients’ answers of the first and the second survey, we applied paired Student’s *t*-test or Wilcoxon singed-rank test for continuous variables, McNemar’s test or Bhapkar’s test for categorial variables in the same subjects. All tests were assessed with 2-sided significance levels of 5%.

Univariate logistic regression was performed including all subjects to determine associations between factors of interest and high perceived risk or high perceived harm. All variables associated with high perceived risk or high perceived harm (*p* ≤ 0.25) in the univariate analysis were included in the multivariable logistic regression analysis. Variables of interest included demographic (gender, age, living in the city of Munich or surroundings), socio-economic factors, such as education level, employment status, income, housing as previously defined [9], COVID-19, IBD and general health related factors (Table S4). Multivariable logistic regression used stepwise elimination, and the final model with no missing in covariates was adjusted for gender and age (in years). In addition, risk assessment (SIRSCO) was evaluated in the final multivariable logistic models where applicable. Interaction and effect modification between considered variables and gender or age were examined in parallel to stepwise elimination. Estimated odds ratio (OR) and 95% CI (confidence interval) were reported. Applying the same procedure, we performed a multivariable logistic regression for IBD patients at working age (20–60 years) to reflect their potentially higher risk of exposure to COVID-19.

To identify changes in estimated risks to acquire COVID-19 infection or harm for a severe course of COVID-19, we repeated the final multivariable logistic models with available data gained from the second survey for the whole cohort as well as for the IBD patients at working age.

Statistical analysis was performed with SAS 9.4 (Statistical Analysis Software, SAS Institute Inc., Cary, NC, USA) and PRISM 8.4 (GraphPad Software).

## 3. Results

### 3.1. Participants and Cohort Description by Age Groups

Of 869 IBD patients identified, 49 patients were excluded (30 adults and 19 children) due to death, duplicates in the database, wrong addresses or moving out of the region, no confirmed IBD diagnosis or monogenetic immunodeficiency disorder (Figure S1). Of the remaining 820 patients, 507 (62%) signed the informed consent and completed the survey, thereof 89/124 (72%) pediatric IBD patients and their parents and 418/696 (60%) adults. We excluded three patients younger than 6 years of age with an unclassified IBD diagnosis, leaving 504 patients aged 6 to 85 years for the analysis of the 1st infection wave. The 2nd survey was completed by 403 (80%) of the 504 previous responders (Figure S1).

Table 1 summarizes characteristics of the total cohort (1st survey) according to 4 age groups (6–20, >20–40, >40–60, and >60 years of age) on demographics, IBD phenotype and medication, potential risk factors and co-morbidities associated with a severe COVID-19 outcome, and influenza vaccination in the last winter season. Many of these items were significantly different between age groups. Crohn’s disease was reported by 58.7%, ulcerative colitis by 35.7% and IBD-u by 4%. Self-reported disease activity differed between age groups. Overall, 11.7% of patients reported moderate and 3.2% severe disease. Disease duration and bowel resections increased with age. Some risk factors associated with severe COVID-19 course were significantly related to older age including smoking, obesity with BMI > 30 kg/m^2^, and co-morbidities such as diabetes, chronic liver, lung or heart disease, stroke, hypertension, renal insufficiency, post-transplantation, and cancer (Table 1 and Table S1). Five patients reported a current pregnancy, none with Human Immunodeficiency Virus (HIV) infection. Almost 20% of the patients received currently either no medication (9.7%) or only 5-Aminosalicylic Acid (5-ASA) (9.5%) for IBD, while 79.6% were treated with at least one immunosuppressive drug, with no difference between age groups. More pediatric patients were treated with immune modulators (IM, mostly azathioprine or methotrexate or combo-therapy (IM plus biologic) compared to adults. Of the total cohort, 40.9% of patients had been vaccinated against influenza in the winter season 2019/20 prior the pandemic with a significant increase in older age groups (Table 1). The vaccination rate increased to 60.2% during the pandemic winter 2020/21 (*p* < 0.0001 for paired comparison).

In the 1st survey, the majority of adults with IBD showed interest in later vaccination against COVID-19, while only 11–14% were not interested. When asked whether healthy household members were interested in COVID-19 vaccination, the percentage of those not interested increased to 20–27%. In the 2nd survey, percentage of those not wishing a vaccination decreased for patients and household members (43.6% to 19.3%, *n* = 296, *p* < 0.0001 and 54.9% to 20.5%, *n* = 264, *p* < 0.0001, respectively, for paired comparison).

### 3.2. Socioeconomic Factors

Most patients lived with one (32.9%), three to four (39.8%) or four and more (9.5%) other household members, while 17.8% lived alone; 37.6% reported living in a family with children. Of the pediatric patients, 15.9% were brought up by a single mother and 1.1% by a single father (1.1%). Housing space per person significantly increased with age, with 28.5% having <30 m^2^, 48% between 30 and 55 m^2^ and 23.7% >55 m^2^ available. Only 7.1% of participants had neither a garden nor a balcony. Twelve years or more of education was reported by 43.0% of IBD patients older than 20 years and by 74.7% of parents of IBD patients. The employment or self-employment rate was 67.5% and 6.6%, respectively, while 20.4% were not working or retired. Of those working, 41.8% had jobs considered to be at increased risk for COVID-19 infection with high number of in-person contacts. Due to the pandemic, 17.5% had experienced shorter work hours, loss of income or loss of employment; 18.2% of the IBD patients were treated differently than fellow colleagues with respect to home-office or stricter measures for keeping social distancing, shorter working hours or being sent on sick leave.

### 3.3. COVID-19 Related Symptoms and Testing for Infection

In the 1st survey, 41.7% of the patients reported at least one symptom possibly related to COVID-19 since the start of the pandemic, with the highest proportion in the youngest (56.7%) and the oldest (47.2%) age group. Symptoms included fever (5.9%), chills (4.9%), muscle pain (12%), infectious rhinitis (16.5%), loss of smell or taste (1.6%), sore throat (14.8%), headache (26.6%), dry persistent cough (5.7%), shortness of breath (3.3%), fatigue (25.3%), and diarrhea with >3 liquid stools per day (25.4%). Testing by nasopharyngeal swab for PCR or serology had been reported by 23.3% and 8% of IBD patients, respectively. Of those, only three were positive by PCR and two tested seropositive. In 99 households (19.7%), one or more members had been tested for COVID-19, with a positive test reported in only two. Serology was performed in 472 of 504 participants prior to the start of the 2nd infection wave, thereof only 2 (0.4%) tested positive, one with known symptomatic PCR positive COVID-19, the second with no symptoms in the past.

In the 2nd survey, further 15 patients (10 males, 11 adults and 4 children, age range 8–63 years, 11 with low and 4 with moderate or high risk for severe course of COVID-19, 14/15 on immunosuppressive medication) reported a proven SARS-CoV-2 infection: 12/15 were diagnosed by a positive PCR test and three by seroconversion prior to vaccination. Only 3/15 were completely asymptomatic, the remaining 12 reported fatigue (9/12), headache (6/12), fever (5/12), chills (4/12), cough (4/12), diarrhea (5/12), and loss of taste/smell (3/12). None of them needed hospitalization. In 8/15 patients, 1 to 3 other household members had been diagnosed with COVID-19. By the time of the 2nd survey, only 39 patients 11.7% of the adult patients had received one (*n* = 18) or two (*n* = 21) vaccinations against COVID-19.

### 3.4. Impact of the Pandemic on IBD-Related Health Care

The pandemic led to medication changes in 4.6% of patients, consisting of dose reduction, change in interval, pausing or omitting ongoing or newly proposed medication. Only 2 (0.4%) patients reported initiating over the counter drugs or supplements hoping for a positive effect. During the first wave of the pandemic, 8.3% of patients required hospitalization for IBD and 5.0% for other medical conditions. While two thirds of the patients (65.5%) kept all regular appointments since the start of the pandemic, 134 (26.7%) reported cancelled outpatient visits, of which 47 had cancelled clinic visits themselves due to concerns of increased risk of infection. During the first infection wave, 86 (17.2%) participants received their medical care via telemedicine (phone, email or both) instead of an outpatient visit. Cancelled or postponed endoscopies or surgery were reported by only 3.8% and 0.4%, respectively.

### 3.5. Impact of the Pandemic on Daily Life

**Figure 1:**
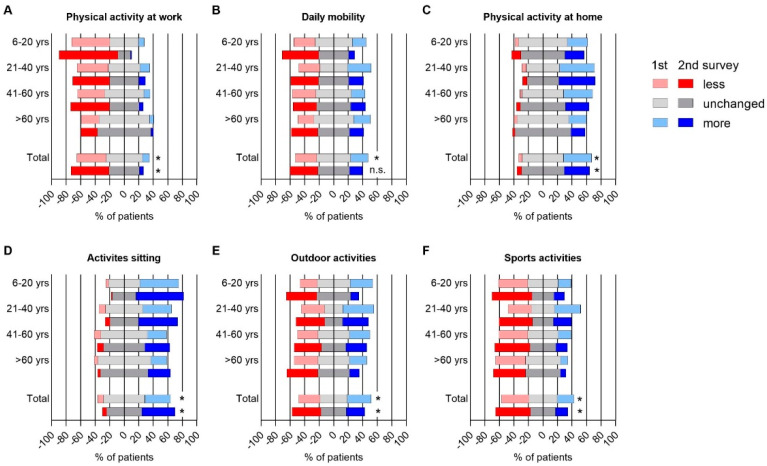
Change in physical activity in IBD patients during the COVID-19 pandemic in first (light colors) and second (dark colors) survey. (**A**) Physical activities at work, (**B**) physical activities regarding daily mobility (e.g., walking, biking to work/school), (**C**) physical activities at home (e.g., dish washing, cleaning, cooking), (**D**) activities sitting, (**E**) leisure time outdoor activities (e.g., gardening, hiking) and, (**F**) sports activities (e.g., cycling, running, strength training), were reported on a 3-scale axis and depicted for four different age groups. The bottom bar represents all IBD patients across different ages. * indicates a statistically significant difference of change in physical activity between four age groups obtained from Pearson’s Chi-square test with *p*-value < 0.05, yrs = years.

Compared to before the pandemic, physical activities decreased in different age groups for work (*p* = 0.002), at home (*p* = 0.003), for daily mobility including walking, biking to school/work (*p* = 0.03), for outdoor activities such as gardening/hiking (*p* = 0.003), for sports activities such as running, fitness and muscle training (*p* = 0.0003), while sitting activities increased (*p* < 0.0001). In the 2nd survey, a further significant reduction of outdoor activities (*p* = 0.019) and sports activities (*p* = 0.0005) was reported, particularly affecting the pediatric patients (Figure 1A–F). Home-schooling affected 81/90 IBD patients <20 years, completely (69%) or partially (31%) and was rated as a negative experience in 31% of children, while 52% of their parents judged home-schooling as worse compared to normal school.

**Figure 2:**
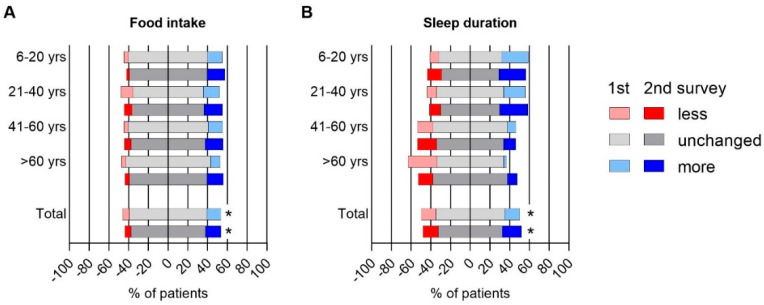
Change in food intake und sleep duration in IBD patients during COVID-19 pandemic in first (light colors) and second (dark colors) survey. (**A**) Daily food intake and (**B**) sleep duration were reported on a 3-scale axis and depicted for 4 different age groups. The bottom bar represents all IBD patients across different ages. * indicates a statistically significant difference of change in food intake and sleep duration between four age groups obtained from Pearson’s Chi-square test with *p*-value < 0.05, yrs = years.

Food intake increased since the start of the pandemic in all age groups, with 15.8% and 18% reporting to eat more in the 1st and the 2nd survey, compared to 6% to eat less (Figure 2A). Accordingly, 22.1% reported an involuntary increase of weight during the pandemic, while involuntary weight loss was less common (4.8%). An increase in coffee consumption since the pandemic was reported in 17.6% of adult IBD patients (total *n* = 416) compared to a decrease in 12% (*p* = 0.003). Inversely, self-reported alcohol consumption decreased in 19.7% but increased in 9.4% (*p* < 0.0001).

Changes of sleep duration during the pandemic were significantly different between age groups, with an increase in children and young adults, but a decrease in elderly people (Figure 2B). Any type of sleep disturbance compared to pre-pandemic times was reported in one of five patients in the two younger age groups and one of four patients in the two older age groups.

### 3.6. Impact of the Pandemic on Self-Perceived Stress and Quality of Life

**Figure 3:**
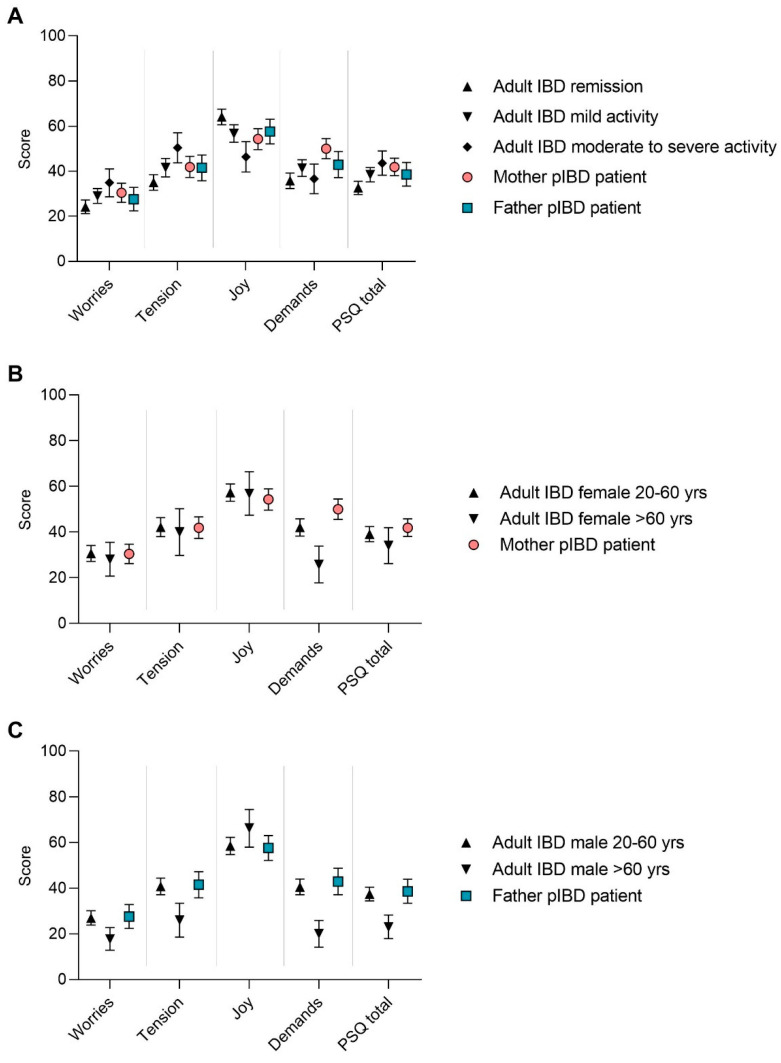
Perceived Stress Questionnaire. Psychological stress was measured in adult IBD patients and in parents of pediatric IBD (pIBD) patients using the short version of the perceived stress questionnaire (PSQ) in the first survey. PSQ scores range 0–100 were given in mean and 95% confidence interval. A high score reflects a high degree of perceived stress (PSQ total), worries, tension, joy or demands. Calculating total PSQ score, joy was transformed into “lack of joy”. Results are shown for adult IBD patients according to disease activity (**A**) and for female (**B**) and male (**C**) IBD patients according to age groups. Mothers and fathers of pIBD patients are shown in relation to adult IBD patients (**A**–**C**), yrs = years.

We analyzed the results of the PSQ score of adult IBD patients according to age group, gender and disease activity and compared them to the scores of parents of pIBD children. Compared to IBD patients in remission (*n* = 188), the PSQ score increased in those with mild (*n* = 157) and moderate to severe disease activity (*n* = 65) (*p* = 0.0053 and *p* = 0.0007, respectively) (Figure 3A). There was no difference between patients with CD and UC. The total PSQ of pIBD mothers (*n* = 84) was similar to adults with moderate to severe disease activity and the average subscore for “demands” was even higher (*p* = 0.0004) (Figure 3A).

Figure 3B shows the PSQ levels for female IBD adults divided into two age groups, those mostly working age 20–60 years (*n* = 160) and those aged >60 years (*n* = 34) and for mothers of IBD-affected children. Younger IBD-patients scored higher (mean PSQ 39.1, 95% CI 35.8–42.4) than older patients (mean PSQ 34.1, 95% CI 26.2–41.9), but the highest stress-level was reported by mothers of affected children (mean PSQ 41.9, 95% CI 38.1–45.8), with significantly higher scores for “demands” compared to females with IBD of similar age (*p* = 0.0072).

In Figure 3C, PSQ is compared between male IBD patients (aged 20–60 years, *n* = 186 and aged >60 years, *n* = 38) and fathers of IBD-affected children. Younger male IBD-patients and pIBD fathers had similar stress levels, while elderly male IBD-patients reported the least stress and highest joy (total PSQ in total *p* < 0.0001, domains “worries”, “tension” and “demands”, *p* = 0.0304, *p* = 0.0005 and *p* < 0.0001, respectively) in all domains and in the total score.

When we applied paired testing in participants of the 1st and 2nd survey, female and male IBD patients aged 20–60 years reported a significant increase in PSQ (mean difference 4.5 points for women and 3.5 for men, *p* = 0.0004 and *p* = 0.0047, respectively) (Figure S3). Participants aged >60 years scored lower compared to the younger participants, with not significant changes between 1st and 2nd part of the pandemic. Mothers of IBD-affected children still scored higher in PSQ than fathers without significant changes between the two time points assessed (Figure S3).

Disease related quality of life (QoL) in adult patients assessed with the validated SIBDQ (range 10–70, higher values indicate better QoL) [13,14] had a mean score of 53.5 (SD = 11). The score was significantly related to disease activity (*p* < 0.001), but not related to age group, type of IBD or duration of disease. Disease-related quality of life in pediatric patients aged 9–17 years was measured with IMPACT III (range 0–100) representing four domains (“well-being”, “emotional functioning”, “social functioning” and “body image”) [15]. PIBD patients reached a mean total score of 75.8 (SD = 10.3), with the lowest values in emotional functioning (mean 63.4, SD = 17.5).

### 3.7. Perceived Risk to Acquire COVID-19 and Perceived Harm for Severe Outcome in Case of Infection

**Figure 4:**
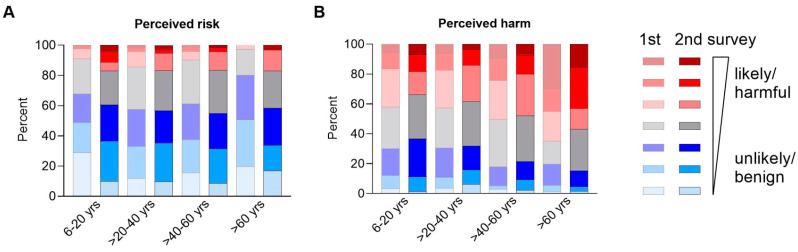
Perceived risk and perceived harm in IBD patients during COVID-19 pandemic in first (light colors, *n* = 501) and second (dark colors, *n* = 389) survey. (**A**) Participants reported perceived risk to be infected with COVID-19 on a 7-scale axis from extremely unlikely to extremely likely. (**B**) Participants reported perceived harm of a COVID-19 infection on a 7-scale axis from extremely benign to extremely harmful; yrs = years.

The patients’ rating of perceived risk to acquire the infection on a scale from 1 to 7, where 1 represents “extremely unlikely” and 7 represents “extremely likely”, is presented according to the four age groups in Figure 4A. A high perceived risk with ratings of >3 was reported by only 36% of IBD patients or their parents (181/501). Patients with high perceived risk were significantly younger and more often females (Table S2).

In contrast, for perceived harm if infected with COVID-19, 75% of IBD patients (378/501) reported a high score (>3) on a scale from 1 to 7 where 1 represents “extremely benign” and 7 represents “extremely harmful” (Figure 4B). Patients rating harm of infection as high were significantly older, but did not differ with respect to gender (Table S3).

In the 2nd survey, the participants estimated their risk to acquire COVID-19 higher compared to the 1st survey (Figure 4A). Perceived harm if infected with COVID-19 tended to be rated lower in paired comparison of 1st and 2nd survey, especially in older patients, who had been vaccinated by the time of the questionnaire (Figure 4B).

#### 3.7.1. Univariate Regression Analysis

We identified several factors, which were significantly related to a high perceived risk to acquire the infection (Table S2). After adjusting for age and gender the following predictors were significantly associated with a higher self-perceived risk for infection: living with more than two household members, having less housing space per person, being employed, working in a high-risk job for potential COVID-19 infection, being obese or overweight, having a longer duration of IBD and a higher SIRSCO index, reporting an increased food intake or an unintended weight change, or postponing a clinic appointment.

Different factors were found to be significantly related to high ratings (>3) for perceived harm in the crude univariate analysis (Table S3). After adjusting for age and gender the following predictors remained significantly associated with perceived harm: lower family income and less housing space per person, avoiding public transport, being obese, active disease and use of immunosuppressive medication, having ≥3 comorbidities associated with increased risk and SIRSCO index ≥2, reporting sleep disturbances, weight change, and cancelled appointment in the IBD clinic cancelled, as well as higher stress scores in all domains and in total.

#### 3.7.2. Multivariable Regression Analysis

**Figure 5:**
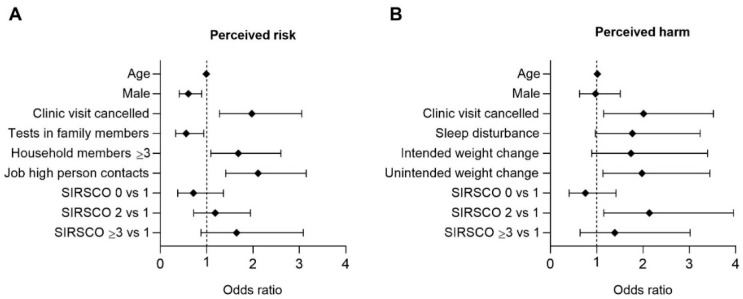
Multivariable logistic regression of patients’ (**A**) perceived risk to acquire COVID-19 (*n* = 495) and (**B**) perceived harm in case of COVID-19 infection (*n* = 497) based on the first survey. Parents of IBD children judged risk and harm on their behalf. SIRSCO (Scoring for increased risk for severe COVID-19 outcome) was described in Methods 2.3 and Figure S2. Mild risk with SIRSCO of 1 was performed as reference category. Other variables of the multivariable logistic regression were specified in Table S4. Odd ratios (OR) with 95% confidence intervals (95% CI) obtained from the multivariable logistic regression model are given.

The final multivariable logistic regression model included variables of interest (Table S4) for perceived risk showed that high perceived risk to acquire the infection was independently inversely associated with male gender (OR 0.6, 95% CI 0.41–0.89, *p* = 0.011) and testing of family members for SARS-CoV-2 infection (OR 0.6; 95% CI 0.33–0.94, *p* = 0.0277), while it was directly related to living with three or more persons in the same household (OR 1.7; 95% CI 1.09–2.60, *p* = 0.0192), at least one household member working in a job with high risk or multiple in-person contacts (OR 2.1, 95% CI 1.41–3.15, *p* = 0.0003), and cancelling clinic visits (OR 2.0; 95% CI 1.28–73.10, *p* = 0.0023) (Figure 5A).

Repeating the analysis with results of the 2nd survey changed with respect to “cancelled clinic visits” and “testing for COVID-19 in house members” which lost its significance (Figure S4A).

The model for perceived harm (first survey) showed that high perceived harm was associated with a higher SIRSCO Index (OR 2.1; 95% CI 1.53–2.95, *p* = 0.0158), cancelling clinic visits (OR 2.0; 95% CI 1.15–3.52, *p* = 0.0144) and unintended weight change (OR 2.0; 95% CI 1.13–3.44, *p* = 0.162) (Figure 5B). Significance was lost for all three items when the model was applied to results from the 2nd survey (Figure S4B).

In a sensitivity analysis for the 1st and 2nd survey limited to IBD patients at working age between 20 and 60 years, showed the same factors were associated with a high perceived risk of infection and perceived harm from infection with COVID-19 (Figure S5A,C). During the 2nd survey, living with three or more household members showed the highest odds for perceived risk of infection. In addition, adult IBD patients at working age with high perceived harm were three times more likely to decrease or avoid the use of public transport (Figure S5B,C).

## 4. Discussion

In this monocentric study, we investigated in a large cohort of IBD patients, ranging from 6–85 years of age, the perceived risk and harm from COVID-19 infection and psychosocial burden after the 1st (1st survey) and during the 3rd infection wave (2nd survey) with respect to age, gender, IBD phenotype, co-morbidities and socioeconomic factors. This wide age range, the repeated assessment during different phases of the pandemic with group to group and intra-individual analyses, the use of validated tools for stress and quality of life and additional questionnaires to mothers and fathers of pIBD patients allowed a differentiated view of the population. We found that pandemic-related restrictions had a particularly negative impact on children and adolescents and their mothers, as well as on IBD patients with moderate to severe active disease, which increased with the duration of the pandemic. Remarkably, elderly adults, particularly males, reported the lowest stress levels in spite of their higher risk for severe COVID-19 outcomes in the 1st survey prior to the availability of vaccinations against COVID-19. Our multivariable model revealed a realistic view of participants on their own risk of infection, which was associated with number of in-person contacts at home or at work. Unexpectedly, male IBD adults judged their risk of infection significantly lower compared to females. Participants with a high perception of harm reported unintended changes of bodyweight, cancelled clinic-visits and co-morbidities or other risk factors reflected by a higher SIRSCO index. Neither age nor gender played a role for high ratings of perceived harm in case of COVID-19 infection after adjusting for SIRSCO index.

Our cohort represents IBD patients across all age groups at an academic center. Half of the cohort was below 40 years of age with a median age at diagnosis of 23 years, which is within the range most IBD patients are diagnosed [31,32]. Compared to other national [33] as well as international cohorts [32] median age was lower due to inclusion of pIBD patients. Fifty-four percent were male with no difference between responders and non-responders. The 80% rate of patients treated with immunosuppressive drugs, the high proportion having perianal involvement, and a bowel resection rate of 23% after a median disease duration of 12 years may indicate a trend for more severe disease in our cohort.

We found that only two IBD patients tested positive for SARS-CoV-2 antibodies after the first infection wave. The seroprevalence in our IBD cohort (0.43%, 95% CI: 0.05–1.55, *p* < 0.0001) was much lower compared to the parallel population-based cohort study (KoCo19) in Munich (1.77%, 95% CI 1.24–2.29) [10], although samples of IBD patients were taken on average 3 months later. The KoCo19 cohort included 5313 participants >14 years of age from 2994 randomly chosen households in Munich and aimed to prospectively assess the seroconversion rate and risk factors for the presence of SARS-CoV-2 antibodies. Age distribution was similar except that 7% of our IBD cohort were children below 14 years of age [10]. We can only speculate whether this difference in seroprevalence was due to a reduced or short-lived immune response with the use of immunosuppressive drugs or whether our IBD patients more strictly adhered to hygiene and social distancing recommendations. Recently, Kennedy et al. showed that infection rates did not differ between infliximab- or vedolizumab-treated IBD patients in the UK, but seroprevalence, seroconversion and the magnitude of anti-SARS-CoV2 antibody reactivity was attenuated in infliximab-treated patients compared to vedolizumab-treated patients [34]. Concomitant immunomodulatory therapies further attenuated anti-SARS-CoV-2 antibody production in infliximab treated patients [34]. Therefore, it seems plausible, that the lower seroprevalence in our cohort may at least in part be attributed to high rates of anti-TNF-treatment or combo-therapy.

Our survey across different age groups revealed an age-dependent impact of the pandemic on daily life. For example, while sleeping time increased in children and young adults because of home-schooling and home-office [35], it decreased in elderly patients. The increase of sleep in children and young adults may reflect that restrictions reduce social jetlag with a shift of sleeping pattern toward evening time [36]. Sleep disturbances have been reported during the pandemic. Among IBD patients, 20 to 25% of patients suffered from any sleep disturbance which is lower compared to adults in Germany (35.7%; 95% Cl, 29.4–42.4%) and international cohorts (38.8%, 95% CI, 37–42%) [37]. Children and adolescents might experience an advantage from flexible sleeping times and increased family time. This may also explain why less children than parents judged homeschooling as negative, at least in this early time of the pandemic. In contrast, parents were more concerned about their children’s education and social interaction and their own role in homeschooling.

The pandemic has impact on eating habits with a higher proportion of IBD patients reporting increased food intake and weight gain during the restrictions, which is in line with observations in other pediatric and adult cohorts globally [35] and in Germany [38]. While a recent review found a decrease of physical activity in adults and children with medical conditions during the pandemic [39], we found physical activity particularly reduced in children and adolescents, while adults were less affected. This observation is alarming, as the positive effects of physical activity in IBD include a reduction of flares and fatigue [40] and long-term effects such as better bone health in the growing child as well as reduction of cardiovascular morbidity and positive effects on mental health are at stake.

Our study was mainly focused on the psychosocial effects of the COVID-19 pandemic. As such, 18.2% of IBD patients reported being treated differently at work by fellow colleagues due to their immune-mediated disease. Furthermore, high rates of cancelled visits to our IBD clinic reflect their feelings of vulnerability due to IBD. Using validated tools for perceived stress and quality of life, we found that participants suffer from psychological stress and reduced quality of life during the COVID-19 pandemic, which increased with duration of the pandemic. The high psychosocial burden even in the first survey was remarkable, since this was recorded in summer 2020 during a phase of relative quiescence of the COVID-19 pandemic in Germany. Several studies show that greater levels of perceived stress are associated with poorer health status and quality of life [41,42]. With increasing disease activity, adult IBD patients report more perceived stress, regardless of the IBD type. Younger IBD patients reported higher levels for perceived stress than older ones. Kujawa et al. showed that especially young adults are at high risk for depression and anxiety during the COVID-19 pandemic [43]. Interestingly, mothers of affected children reported a significantly higher perceived stress compared to women with IBD of similar age. The stress level of pIBD mothers corresponds to that of an adult IBD patient with moderate to severe active disease. Particularly, pIBD mothers suffer from the demands placed on them. It has been described that it is a great challenge for parents, especially during the COVID-19 pandemic, to find the balance between their parental role and their work life [44]. This may be further aggravated in our subgroup of single pIBD mothers who carry the burden alone. It is also well known that chronic conditions like pIBD additionally represent a high-impact stress for parents [45,46,47]. Mothers of pIBD children report higher levels of psychological distress in comparison to healthy controls [48] and take on numerous tasks in disease management [49]. The significant increase in PSQ score with the duration of the pandemic confirms the findings of Calvano et al. who reported of increasing stress levels especially in parents during the pandemic [50].

In parallel to the PSQ, increasing disease activity reduced quality of life in the SIBDQ in adult patients. Patients in remission however, report a good quality of life. Similarly, Yu et al. found a good health-related quality of life in up to 85% of IBD patients (67–80% in remission) during the first wave of the COVID-19 pandemic [51]. Our IBD-affected children experienced a reduction in their quality of life during the COVID-19 pandemic, especially regarding emotional and social functioning as well as general well-being, assessed by IMPACT III. Martinelly et al. also used the IMPACT III in IBD-affected children with no difference in total score compared to our cohort [52]. The increasing reduction of emotional and social functioning in our cohort is probably due to the length of the lockdown during the 2nd and 3rd infection wave. Our questionnaires were collected over a period of almost one-year duration of the pandemic, while Martinelli et al. used data from the early beginning of the pandemic in spring 2020.

Analysis of perceived risk and harm of COVID-19 infection revealed a realistic judgement regarding their risk to acquire COVID-19 mostly related to in-person contacts in school, at work or at home. In the 2nd survey, the patients’ perception of risk to acquire COVID-19 adequately increased compared to the 1st wave. Male patients judged their risk significantly lower compared to female patients, even when adjusted for working situation. While males and females are at equivalent risk of infection, male sex is associated with complicated COVID-19, reflected by more intensive care admissions and increased death rates. The lack of metadata on possible sex-based differences in comorbidities may drive some of the differences that could be found with adjustments for these [53]. In our cohort, participants with a high perception of complications in case of COVID-19 reported unintended weight change, cancelled clinic-visits, avoided public transport and higher SIRSCO index. Neither age nor gender played a role for a higher rating of perceived harm after adjustment, which may indicate that the SISCO-score appropriately captured the possible confounders for severe COVID-19 outcome.

We consistently observed age-dependent differences regarding the general well-being in our cohort. Older persons reported less negative and more positive emotions in their current lives during the pandemic. This relative age advantage cannot be explained by risk denial, as older age was found to be positively correlated with perceived risk of COVID-19 in the same study, suggesting the awareness of older adults regarding their heightened risks compared to younger adults [54]. Younger adults in studies early during the pandemic had more COVID-19 worries and more behavioral changes than older men but not older women, with greater resilience in elderly [55]. Better mental health reported in older adults despite their realistic perception of higher risk for a severe course of COVID-19 infection has been linked to fewer non-COVID-19 stressors like work environment and higher levels of coping efficacy compared to younger adults. Work and family stressors for younger and middle-aged adults were particularly evident, while older adults had less difficulties to avoid situations with in-person contacts [56].

The main strengths of this study include the large age-span of the representative IBD cohort, the high response rate, few missing data for the different items in spite of the extensive questionnaires, the long follow-up time over almost 1 year with repeated assessments and the standardized assessment of psychological stress and quality of life for IBD-patients and fathers and mothers of pIBD patients, reflecting the different views in the society from school to work and up to retirement. Collection of data on many fields of interest allowed logistic regression models to adjust for confounding factors. As limitations, we consider that the first survey was collected over 3 months and mainly recorded in summer 2020 when infections rates were low in Germany. Therefore, our patient-reported data are subject to recall bias that may have underestimated the prevalence of stress and possible COVID-19 symptoms.

In summary, we found a high degree of worries and stress in our IBD cohort of mixed ages, which increased with the duration of the COVID-19 pandemic. PIBD patients and their families seem to be mostly affected. Furthermore, the pandemic substantially affects weight gain, food intake and physical activity. IBD teams should be encouraged to start early intervention programs to prevent long-term negative effects on mental and physical health in this vulnerable population. IBD patients are in need of valid information regarding their risks due to IBD and related medication. Male patients are at risk to underestimate their risk from COVID-19 and should be targets of educational communication strategies to promote social distancing and finally vaccinations. The lower sero-prevalence prior to the 2nd infection wave compared to the general population may be partly attributed to IBD-medication. Therefore, studies to determine the immune response following COVID-19 immunization and the formation of antibodies after COVID-19 illness are urgently warranted.

## Figures and Tables

**Table 1:** Patient characteristics of the total cohort (*N* = 504) in four age groups.

Factors, *n* (%)	All Patients	6–20 Years	>20–40 Years	>40–60 Years	>60 Years	*p*-Value ^f^
	*N* = 504 (100%)	90 (18%)	167 (33%)	175 (35%)	72 (14%)	
**Male sex**	272 (54.0)	51 (56.7)	88 (52.7)	95 (54.3)	38 (52.8)	0.94
**Age at inclusion** in y, median (IQR)	40 (27–54)	15 (12–17)	33 (28–36)	50 (45–55)	68 (63–74)	**<0.001**
**Age at diagnosis** in y, median (IQR), ***N* = 491**	23 (15–33)	10 (7–13)	21 (16–27)	30 (21–39)	48 (28–57)	**<0.001**
**Disease duration** in y, median (IQR), ***N* = 491**	12 (5–21)	4 (1–7)	10 (6–15)	18.5 (11.5–27.5)	23 (12–36)	**<0.001**
**Influenza vaccination season 2019/20**	204 (40.5)	33 (36.7)	60 (35.9)	59 (33.7)	41 (56.9)	**0.008**
**BMI categories** ^a^						**<0.001**
Underweight	40 (8.0)	26 (29.2)	10 (6.0)	3 (1.7)	1 (1.4)	
Normal weight	275 (54.7)	54 (60.7)	109 (65.3)	82 (46.9)	30 (41.7)	
Overweight	130 (25.8)	8 (9.0)	34 (20.4)	57 (32.6)	31 (43.1)	
Obesity	58 (11.5)	1 (1.1)	14 (8.4)	33 (18.9)	10 (13.9)	
**IBD type**						**0.005**
Crohn’s Disease	296 (58.7)	44 (48.9)	100 (59.9)	110 (62.9)	42 (58.3)	
Ulcerative colitis	180 (35.7)	34 (37.8)	55 (32.9)	61 (34.9)	30 (41.7)	
IBD-u	20 (4.0)	10 (11.1)	8 (4.8)	2 (1.1)	0 (0.0)	
**Disease activity**						**0.020**
Remission	230 (45.7)	40 (44.9)	92 (55.1)	71 (40.6)	27 (37.5)	
Mild	198 (39.4)	39 (43.8)	54 (32.3)	80 (45.7)	25 (34.7)	
Moderate	59 (11.7)	8 (9.0)	16 (9.6)	19 (10.9)	16 (22.2)	
Severe	16 (3.2)	2 (2.2)	5 (3.0)	5 (2.9)	4 (5.6)	
**Any surgery due to IBD**	185 (36.7)	16 (17.8)	51 (30.5)	80 (45.7)	38 (52.8)	**<0.001**
**Any bowel resection**	116 (23.0)	6 (6.7)	26 (15.6)	57 (32.6)	27 (37.5)	**<0.001**
**SIRSCO** ^b^ **Index, *N* = 504**						**<0.0001**
0 = no increased risk	58 (11.5)	15 (16.7)	22 (13.2)	18 (10.3)	3 (4.2)	
1 = mild	249 (49.4)	59 (65.6)	104 (62.3)	73 (41.7)	13 (18.1)	
2 = moderate	116 (23.0)	16 (17.8)	32 (19.2)	49 (28.0)	19 (26.4)	
3–4 = severe	66 (13.1)	0 (0.0)	9 (5.4)	32 (18.3)	25 (34.7)	
≥5 = very severe	15 (3.0)	0 (0.0)	0 (0.0)	3 (1.7)	12 (16.7)	
**Any drug for non-IBD disease**	198 (39.5)	14 (15.9)	47 (28.1)	85 (48.6)	52 (72.2)	**<0.001**
**IBD associated medication**						
None	49 (9.7)	3 (3.3)	18 (10.8)	21 (12.0)	7 (9.7)	0.14
Only 5-ASA	48 (9.5)	10 (11.1)	15 (9.0)	15 (8.6)	8 (11.1)	0.87
Any 5-ASA	164 (32.5)	42 (46.7)	48 (28.7)	47 (26.9)	27 (37.5)	**0.005**
Any Immune modulator ^c^ (IM)	67 (13.3)	37 (41.1)	15 (9.0)	8 (4.6)	7 (9.7)	**<0.001**
Any biologic ^d^/Jak-Inhibitor	350 (69.4)	60 (66.7)	120 (71.9)	127 (72.6)	43 (59.7)	0.19
Any current corticosteroids	64 (12.7)	8 (8.9)	16 (9.6)	22 (12.6)	18 (25.0)	**0.006**
Any immunosuppressive medication incl. biologics, IM, corticosteroids	401 (79.6)	74 (82.2)	133 (79.6)	138 (78.9)	56 (77.8)	0.9
Combo-therapy ^e^ +/− 5-ASA	33 (6.5)	25 (27.8)	7 (4.2)	1 (0.6)	0 (0.0)	**<0.001**
Any supplements incl. probiotics, vitamins, over the counter drugs	383 (76.0)	76 (84.4)	121 (72.5)	131 (74.9)	55 (76.4)	0.19

Results were presented in median (IQR) for continuous variables including age at inclusion (years), age at diagnosis (years), disease duration (years), and in frequency (n) and column percentage (%) for categorial variables. ^a^ BMI categories refer to WHO criteria for adults ≥20 years and CDC-WHO BMI z-score for children <20 years. ^b^ SIRSCO (Scoring for increased risk for severe COVID-19 outcome) was described in Section 2.3 and Figure S2. ^c^ Immunomodulators (IM): azathioprine, mercaptopurine, methotrexate or cyclosporine. ^d^ Biologics: infliximab, adalimumab, golimumab, vedolizumab, and ustekinumab. JAK inhibitor: tofacitinib. ^e^ Combo-therapy: any biologic and IM or dual-biologics or biologic and JAK-inhibitor. ^f^ *p*-values obtained by Mann–Whitney-*U*-test for continuous variables including age at inclusion (years), age at diagnosis (years), disease duration (years), while Pearson’s Chi-square test, or Fisher’s exact test for categorial variables as appropriate. Bold *p*-values indicate significant differences in the proportion of respective factors in 4 age groups with a *p*-value ≤ 0.05.

## Data Availability

Individual data cannot be shared for ethical/privacy reasons. Analyzed and anonymized data are available on request.

## Supplementary Materials

The following are available online at https://www.mdpi.com/article/10.3390/jcm10184124/s1, Figure S1: Flow chart of participants in the cohort study, Figure S2: SIRSCO index, Figure S3: Matched PSQ of pIBD mothers and fathers as well as IBD patients, Figure S4: Multivariable logistic regression based on second survey, Figure S5: Multivariable logistic regression patients aged 20 to 60 years of age, Table S1: Patient characteristics of the total cohort (*N* = 504) in four age groups (continued), Table S2: Univariate analysis of factors associated with high perceived risk of acquiring COVID-19 infection, *N* = 501, Table S3: Univariate analysis of factors associated with high perceived harm in case of COVID-19 infection, *N* = 501, Table S4: Considered variables for multivariable logistic regression.
