# Supplementary material for: Following Pediatric and Adult IBD Patients through the COVID-19 Pandemic: Changes in Psychosocial Burden and Perception of Infection Risk and Harm over Time
Source: J Clin Med. 2021 Sep 13;10(18):4124. doi: 10.3390/jcm10184124 (PMC8464949; doi:10.3390/jcm10184124)
Supplement: Supplementary file 1 [file jcm-10-04124-s001.zip › jcm-1356919-supplementary.pdf]

Supplementary Information for:

Following pediatric and adult IBD patients through the COVID-19 pandemic: changes in psychosocial burden and perception of infection risk and harm over time

Leandra Koletzko, Elisabeth Klucker, Thu Giang Le Thi, Simone Breiteneicher, Raquel Rubio-Acero, Lukas Neuhaus, Renee Stark, Marie Standl, Michael Hölscher, Helga Török, Sibylle Koletzko and Tobias Schwerd

The PDF file includes:

Supplementary Figure S1	Flow chart of participants in the cohort study
Supplementary Figure S2	SIRSCO index
Supplementary Figure S3	Matched PSQ of PIBD mothers and fathers as well as IBD patients
Supplementary Figure S4	Multivariable logistic regression based on second survey
Supplementary Figure S5	Multivariable logistic regression patients aged 20 to 60 years of age
Supplementary Table S1	Patient characteristics of the total cohort (N=504) in four age groups (continued)
Supplementary Table S2	Univariate analysis of factors associated with high perceived risk of acquiring COVID-19 infection, N=501
Supplementary Table S3	Univariate analysis of factors associated with high perceived harm in case of COVID-19 infection, N=501
Supplementary Table S4	Considered variables for multivariable logistic regression

Supplementary Figure S1

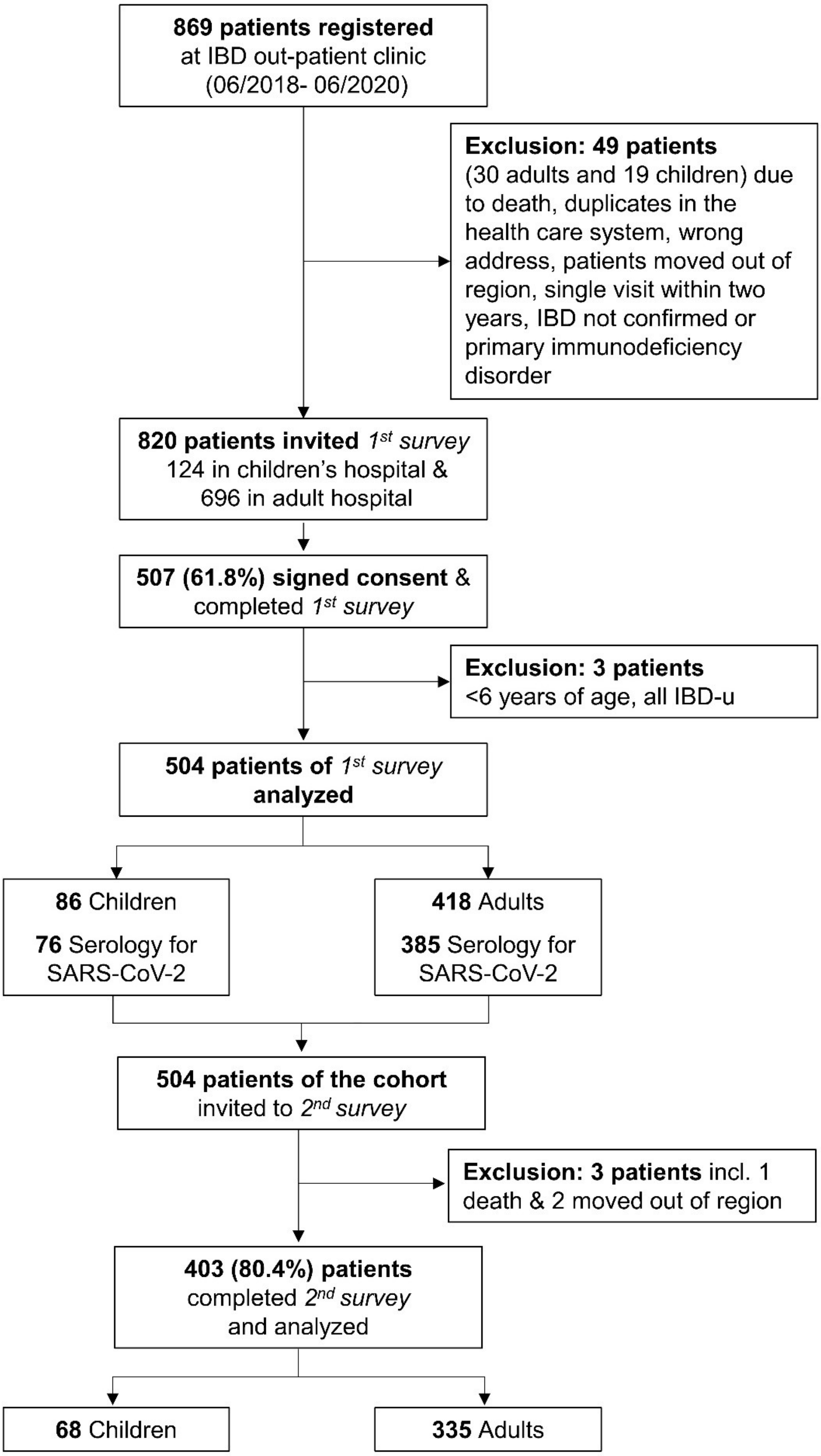

Fig. S1: Flow chart of participants in the first and second survey of the COVID-19 cohort study.

Supplementary Figure S2

I. High-risk comorbidities associated with severe COVID-19 illness:

1. Diabetes (type I or type II)
2. Chronic liver disease
3. Chronic lung disease
4. Chronic heart disease
5. Arterial hypertension
6. Rheumatic diseases
7. Stroke
8. Cancer
9. Renal insufficiency
10. Overweight / Obesity or BMI ≥30 kg/m^2
11. HIV

1 point, if one comorbidity
2 points, if two comorbidities
3 points, if ≥3 comorbidities

**II. Transplantation (organ, hematopoietic)**	+ 2 points
**III. Treatment with any immunosuppressive medication including biologics, immuno-modulator or steroids**	+ 1 point
**IV. Age >70 years**	+ 1 point

SIRSCO Total Score:

- 0 points, patient has **no increased risk**.
- 1 point, patient has **mild risk**.
- 2 points, patient has **moderate risk**.
- 3-4 points, patient has **severe risk**
- ≥ 5 points, patient has **very severe risk**.

Fig. S2: Scoring for increased risk for severe COVID-19 outcome SIRSCO index. Based on information patients reported on their currently used medications, known comorbidities, weight and height, we calculated an individual risk score for severe illness from COVID-19. Comorbidities were selected based on information provided by the Robert Koch Institute, a public health institute in Germany (www.rki.de). Chronic liver disease included autoimmune hepatitis, cholangitis (PSC) or other chronic liver disease, chronic lung disease included asthma, COPD, or other chronic lung diseases, chronic heart disease included coronary heart disease, heart insufficiency, cardiac arrhythmia or peripheral arterial disease. Other comorbidities reported by patients on skin allergy, psoriasis, allergies of the respiratory tract, autoimmune disease, other comorbidities / other chronic diseases were excluded in the consideration of high-risk comorbidities. Based on the total score, patients were stratified in 5 risk groups for severe illness from COVID-19.

Supplementary Figure S3

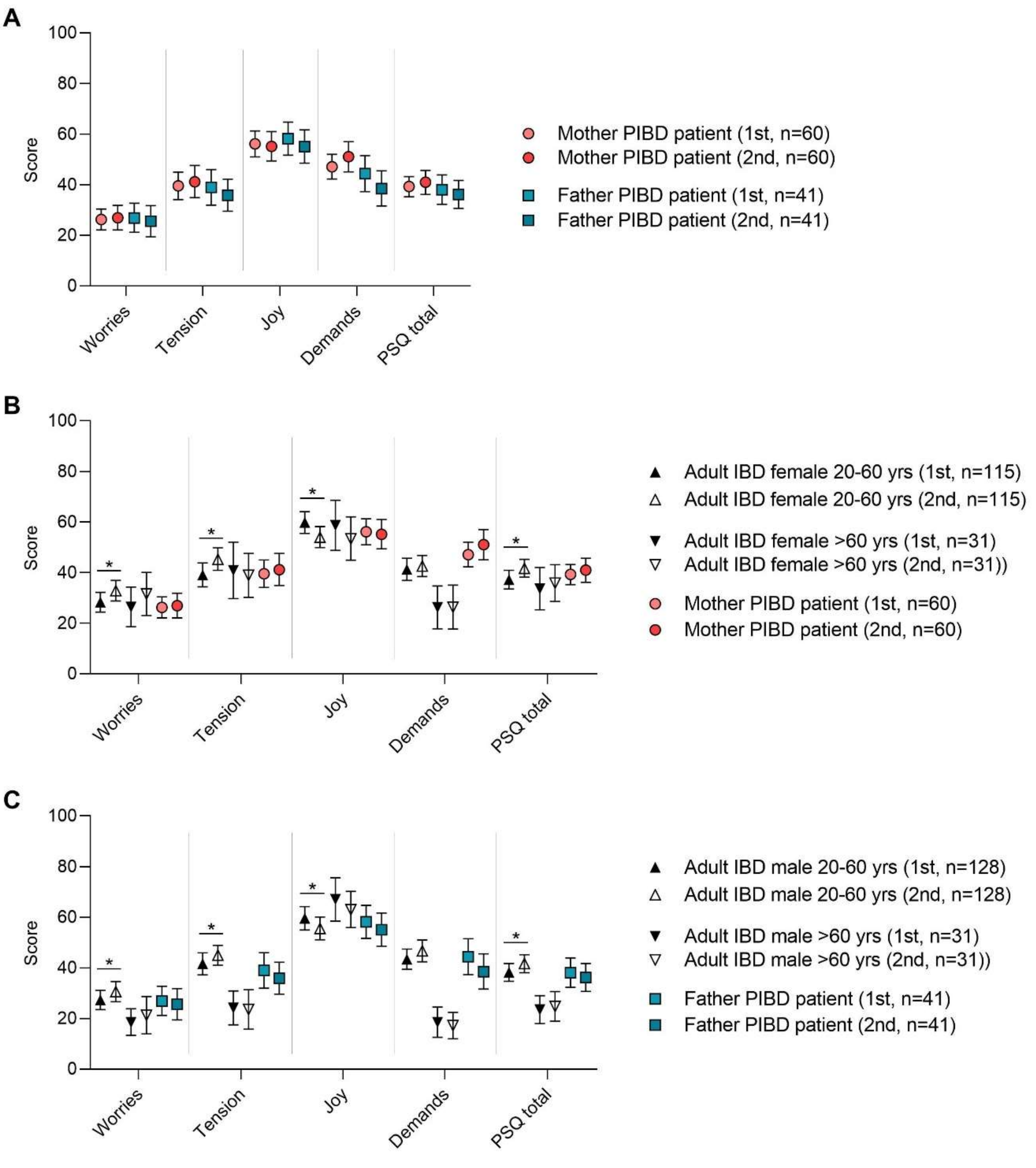

Fig. S3 Perceived Stress Questionnaire (PSQ) in first (1st) and second (2nd) survey during the COVID-19 pandemic. Psychological stress was measured in adult IBD patients and in parents of pediatric IBD (PIBD) patients using the short version of the perceived stress questionnaire (PSQ). PSQ scores range 0-100 were given in mean and 95% confidence interval. A high score reflects a high degree of perceived stress (PSQ total), worries, tension, joy, or demands. Calculating total PSQ score, joy was transformed into “lack of joy”. Results are shown for mothers and fathers of PIBD patients **(A)** and for female **(B)** and male **(C)** IBD patients according to age groups from first (light colors and filled symbols) and second (dark colors and open symbols) survey. Mothers and fathers of PIBD patients are shown in relation to adult IBD patients **(B, C)**.

Supplementary Figure S4

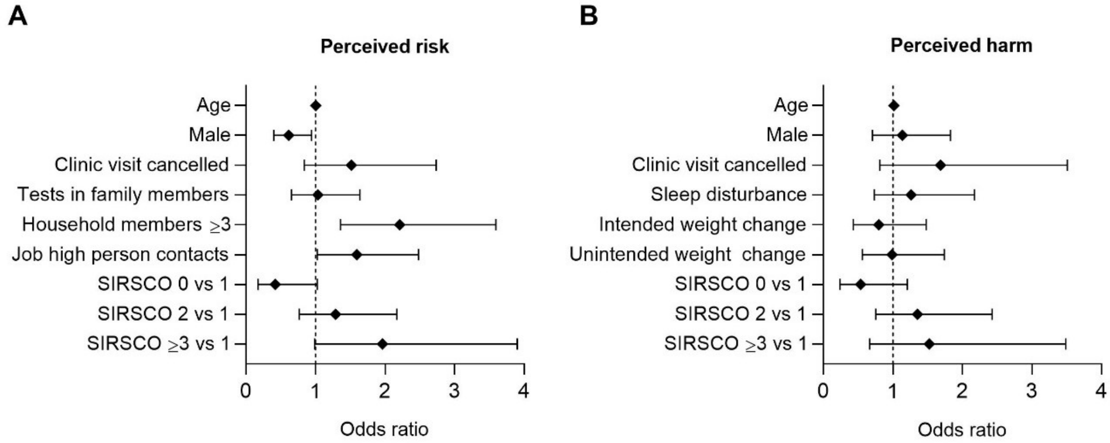

Fig. S4: Multivariable logistic regression of patients' **(A)** perceived risk to acquire COVID-19 (n = 387) and **(B)** perceived harm in case of infection (n = 386) in the second survey. SIRSCO (Scoring for increased risk for severe COVID-19 outcome) was described in Methods 2.3 and Figure S2. Mild risk with SIRSCO of 1 was performed as reference category. Other variables of the multivariable logistic regression were specified in Table S5. Odd ratios (OR) with 95% confidence intervals (95% CI) obtained from the multivariable logistic regression model are given.

Supplementary Figure S5

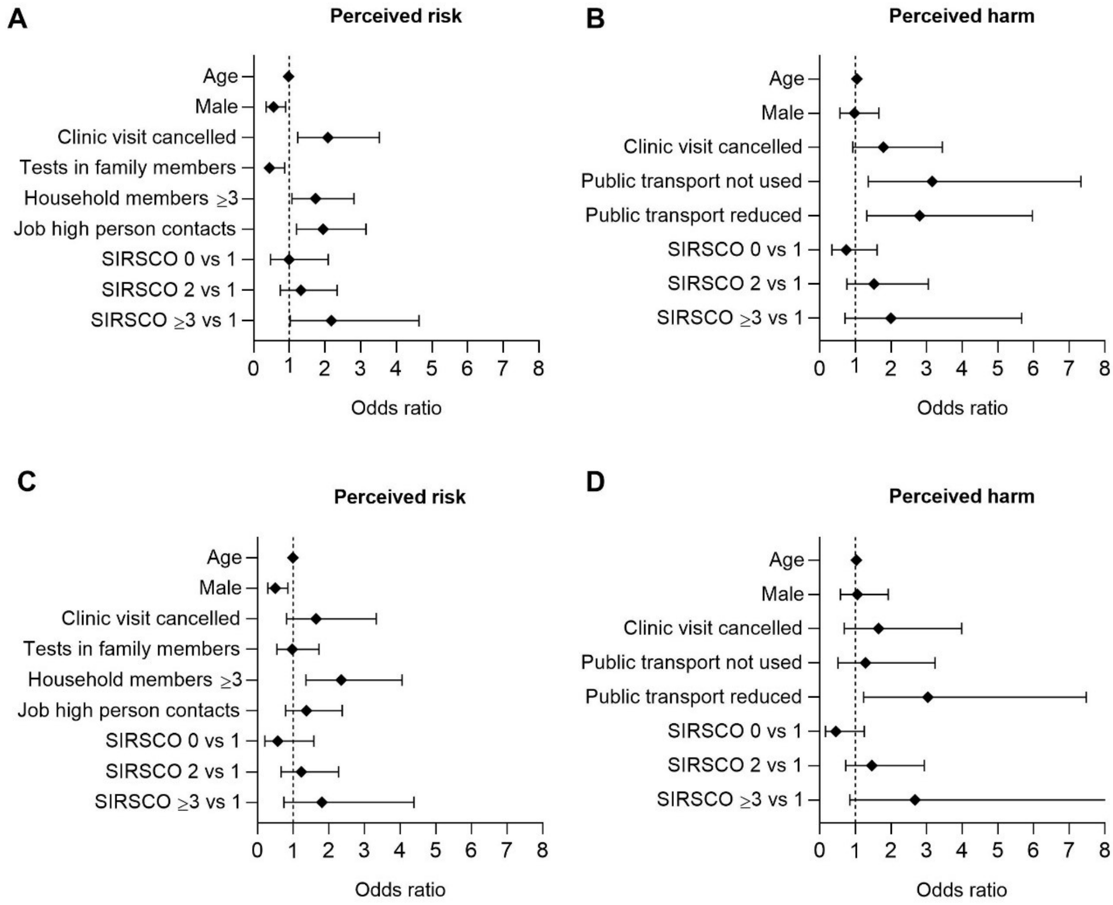

Fig. S5: Multivariable logistic regression of patients' (aged 20-60 years) perceived risk to acquire COVID-19 and perceived harm in case of infection in the first **(A and B)** and the second **(C and D)** survey. **(A)** Perceived risk, first survey (n=340). **(B)** Perceived harm, first survey (n=343). **(C)** Perceived risk, second survey (n=255). **(D)** Perceived harm, second survey (n=255). Patients between 20 to 60 years of age were included into the analysis. SIRSCO (Scoring for increased risk for severe COVID-19 outcome) was described in Methods 2.3 and Figure S2. Mild risk with SIRSCO of 1 was performed as reference category. Other variables of the multivariable logistic regression were specified in Table S5. Odd ratios (OR) with 95% confidence intervals (95% CI) obtained from the multivariable logistic regression model are given.

Supplementary Table S1: Patient characteristics of the total cohort (N=504) in four age groups (continued)

Factors, n (%)	All patients N=504 (100%)	6 – 20 y 90 (18%)	>20 – 40 y 167 (33%)	>40 – 60 y 175 (35%)	>60 y 72 (14%)	p-value[f]
**Country of birth**, Germany	451 (89.5)	82 (91.1)	145 (86.8)	157 (89.7)	67 (93.1)	0.48
**Place of living**, Munich	175 (34.7)	23 (25.6)	69 (41.3)	59 (33.7)	24 (33.3)	0.083
**Smoking >1 year/lifetime**	221 (45.9)	10 (14.7)	62 (37.1)	104 (59.8)	45 (62.5)	**<0.001**
**First degree relative with IBD**	67 (13.3)	14 (15.6)	22 (13.2)	22 (12.6)	9 (12.5)	0.98
**Crohn's disease phenotype**[a]**, N=290**						0.34
L1 ileo-cecal	63 (21.7)	5 (11.6)	23 (23.7)	25 (22.9)	10 (24.4)	
L2 colonic	42 (14.5)	3 (7.0)	16 (16.5)	20 (18.3)	3 (7.3)	
L3 Ileo-colonic	160 (55.2)	30 (69.8)	50 (51.5)	55 (50.5)	25 (61.0)	
L4 only (upper GI incl. jejunum)	25 (8.6)	5 (11.6)	8 (8.3)	9 (8.3)	3 (7.3)	
**B2 stricturing disease**[b]**, N=295**	66 (22.4)	6 (13.6)	20 (20.0)	28 (25.7)	12 (28.6)	0.46
**Perianal involvement**[c]**, N=295**	135 (45.8)	16 (36.4)	49 (49.0)	55 (50.5)	15 (35.7)	0.13
**Ulcerative colitis phenotype**[d]**, N=142**						0.16
E1 Proctitis	20 (14.1)	3 (12.0)	3 (6.5)	8 (17.0)	6 (25.0)	
E2 Left sided	23 (16.2)	1 (4.0)	8 (17.4)	9 (19.2)	5 (20.8)	
E3 Extensive	99 (69.7)	21 (84.0)	35 (76.1)	30 (63.8)	13 (54.2)	
**Co-morbidities with increased risk for severe COVID-19 outcome**						
Diabetes (Type I or Type II), N=504	27 (5.4)	2 (2.2)	0 (0.0)	15 (8.6)	10 (13.9)	**<0.001**
Chronic lung disease, N=504	47 (9.3)	8 (8.9)	12 (7.2)	15 (8.6)	12 (16.7)	0.13
Chronic liver disease, N=504	45 (8.9)	6 (6.7)	13 (7.8)	21 (12.0)	5 (6.9)	0.36
Chronic heart disease, N=418	19 (4.6)	0 (0.0)	1 (0.6)	4 (2.3)	14 (19.4)	**<0.001**
Arterial hypertension, N=418	71 (17.0)	0 (0.0)	6 (3.6)	37 (21.1)	28 (38.9)	**<0.001**
Renal insufficiency, N=418	19 (4.5)	0 (0.0)	5 (3.0)	9 (5.1)	5 (6.9)	0.53
Rheumatic disease, N=418	47 (11.2)	0 (0.0)	7 (4.2)	21 (12.0)	19 (26.4)	**<0.001**
Stroke, N=418	5 (1.2)	0 (0.0)	1 (0.6)	1 (0.6)	3 (4.2)	0.089
Malignancy, N=418	15 (3.6)	0 (0.0)	4 (2.4)	3 (1.7)	8 (11.1)	**0.002**
Obesity BMI ≥ 30 kg/m^2, N=504	58 (11.5)	1 (1.1)	14 (8.4)	33 (18.9)	10 (13.9)	**0.0001**
**Categories of high-risk comorbidity, N=504**						**<0.0001**
None	290 (57.5)	73 (81.1)	116 (69.5)	82 (46.9)	19 (26.4)	
One high-risk comorbidity	132 (26.2)	17 (18.9)	41 (24.5)	54 (30.9)	20 (27.8)	
Two high-risk comorbidities	46 (9.1)	0 (0.0)	8 (4.8)	23 (13.1)	15 (20.8)	
≥3 high-risk comorbidities	36 (7.1)	0 (0.0)	2 (1.2)	16 (9.1)	18 (25.0)	
**Transplantation (organ, hematopoietic stem cell), N=504**	9 (1.8)	0 (0.0)	1 (0.6)	5 (2.9)	3 (4.2)	0.092

Results were presented in frequency (n) and column percentage (%).

[a b c d e] *Phenotypes were categorized based on self-assessment of participants on IBD disease course.*

[f] *P-values obtained from Pearson's Chi-square test or Fisher's exact test as appropriate.*

Bold p-values indicate significant differences in the proportion of respective factors in 4 age groups with a p-value <0.05.

Supplementary Table S2: Univariate analysis of factors associated with high perceived risk of acquiring COVID-19 infection, N=501

Factors, N (row %)	Total cohort, N=501	Low perceived risk (rating ≤ 3) n=320 (64%)	High perceived risk (rating > 3) n=181 (36%)	p-value[a]	Crude OR (95%CI)	p-value[b]	OR adjusted for age and gender (95%CI)	p-value[c]
**Age in years, median (IQR)**	40 (27-54)	41 (26-56)	38 (28-50)	**<0.001**	0.98 (0.98 - 0.99)	**<0.0001**		
**Age groups**				**0.006**				
6 – 20	90	61 (68%)	29 (32%)		1.94 (1.13 – 3.28)	**0.01**		
>20 – 40	167	96 (57%)	71 (43%)		3.01 (2.1 - 4.36)	**<0.0001**		
>40 – 60	173	106 (61%)	67 (39%)		2.57 (1.84 - 3.65)	**<0.0001**		
>60	71	57 (80%)	14 (20%)		1			
**Gender**				**0.004**				
Female	231	132 (58%)	99 (43%)		1			
Male	270	188 (70%)	82 (31%)		0.58 (0.42 - 0.8)	**0.0009**		
**Living in Munich**				0.45				
Yes	174	115 (66%)	59 (34%)		1		1	
No	327	205 (63%)	122 (37%)		1.16 (0.82 - 1.66)	0.41	1.16 (0.82 - 1.67)	0.40
**Housing space per person**				**0.015**				
≤30m2 - small space	140	87 (62%)	53 (38%)		1		1	
30m2 to 55m2 – medium space	239	141 (59%)	98 (41%)		1.14 (0.78 - 1.69)	0.50	1.13 (0.76 - 1.68)	0.55
>55m2 - large space	118	88 (75%)	30 (25%)		0.56 (0.37 - 0.85)	**0.006**	0.61 (0.40 - 0.94)	**0.02**
**Number of household members**				**0.009**				
1 or 2	254	176 (69%)	78 (31%)		1		1	
≥3	245	142 (58%)	103 (42%)		1.64 (1.19 - 2.26)	**0.003**	1.68 (1.19 - 2.40)	**0.004**
**Employment status**				0.12				
Employed	314	186 (59%)	128 (41%)		1.73 (1.23 - 2.45)	**0.002**	1.51 (1.03 – 2.21)	**0.03**
Self-employed	31	22 (71%)	9 (29%)		1.03 (0.55 - 1.86)	0.92	1.09 (0.57 – 2.02)	0.79
Not working	95	68 (72%)	27 (28%)		1		1	
**Net income per household**				0.75				
≤2500 €	105	62 (59%)	43 (41%)		1.28 (0.81 - 2.02)	0.28	1.28 (0.80 – 2.03)	0.31
2500 to 4000 €	109	71 (65%)	38 (35%)		0.99 (0.69 - 1.43)	0.96	1.02 (0.70 – 1.47)	0.94
4000 to 6000 €	114	69 (61%)	45 (39%)		1.21 (0.88 - 1.66)	0.25	1.30 (0.94 – 1.79)	0.12
>6000 €	77	50 (65%)	27 (35%)		1		1	
**Level of education**				0.19				
<12 years education	201	136 (68%)	65 (32%)		1		1	
≥12 years education	283	175 (62%)	108 (38%)		1.29 (0.92 - 1.82)	0.14	1.25 (0.88 – 1.80)	0.22
**High-risk job for COVID-19 infection**				**0.0002**				
Yes	209	114 (55%)	95 (45%)		1.99 (1.44 - 2.76)	**<0.0001**	1.88 (1.35 – 2.61)	**0.0002**
No	292	206 (71%)	86 (29%)		1		1	
**Public transport usage reduced**				0.07				
No	59	43 (73%)	16 (27%)		1		1	
Yes	291	174 (60%)	117 (40%)		1.81 (1.01 - 3.38)	**0.05**	1.71 (0.95 – 3.21)	0.08
Does not apply to me	148	101 (68%)	47 (32%)		1.25 (0.69 - 2.36)	0.47	1.21 (0.67 – 2.29)	0.55

Factors, N (row %)	Total cohort, N=501	Low perceived risk (rating ≤ 3) n=320 (64%)	High perceived risk (rating > 3) n=181 (36%)	p-value[a]	Crude OR (95%CI)	p-value[b]	OR adjusted for age and gender (95%CI)	p-value[c]
**Temporary incapacity to work since 3/20**				**0.007**				
No	304	203 (67%)	101 (33%)		1		1	
Yes, due to IBD or other reasons	111	58 (52%)	53 (48%)		1.84 (1.22 - 2.77)	**0.004**	1.80 (1.18 – 2.74)	**0.006**
**BMI**				0.16				
Underweight	40	25 (62%)	15 (38%)		1.19 (0.6 - 2.29)	0.60	1.00 (0.50 – 1.94)	0.99
Normal weight	275	183 (67%)	92 (33%)		1		1	
Overweight	128	83 (65%)	45 (35%)		1.08 (0.82 - 1.42)	0.59	1.31 (0.98 – 1.76)	**0.07**
Obesity	57	29 (51%)	28 (49%)		1.92 (1.4 - 2.63)	**<.0001**	2.22 (1.59 – 3.10)	**<0.0001**
**IBD Type**				0.41				
Crohn's Disease	295	183 (62%)	112 (38%)		1.17 (0.86 - 1.6)	0.33	1.13 (0.82 – 1.56)	0.45
Ulcerative Colitis / IBD-u	198	130 (66%)	68 (34%)		1		1	
**Disease activity (self-assessment)**				0.23				
Remission	228	146 (64%)	82 (36%)		1		1	
Mild activity	197	120 (61%)	77 (39%)		1.14 (0.82 - 1.61)	0.44	1.16 (0.83 – 1.64)	0.39
Moderate / severe activity	75	54 (72%)	21 (28%)		0.69 (0.46 - 1.03)	0.07	0.75 (0.50 – 1.13)	0.17
**Duration of IBD (to 2020)**				0.66				
0 to 5 years	126	86 (68%)	40 (32%)		1		1	
6 to 10 years	93	56 (60%)	37 (40%)		1.42 (0.89 - 2.3)	0.15	1.64 (1.01 – 2.69)	**0.049**
11 to 20 years	147	94 (64%)	53 (36%)		1.21 (0.8 - 1.86)	0.37	1.80 (1.15 – 2.88)	**0.01**
over 20 years	122	77 (63%)	45 (37%)		1.26 (0.83 - 1.92)	0.28	2.59 (1.57 – 4.37)	**0.0003**
**Influenza vaccination**				0.49				
Yes	203	133 (66%)	70 (34%)		0.88 (0.63 - 1.22)	0.44	0.85 (0.60 – 1.20)	0.36
No	293	183 (62%)	110 (38%)		1		1	
**Any immunosuppressive medications used incl. IM, biologics, steroids**				0.26				
Yes	399	250 (63%)	149 (37%)		1.3 (0.91 - 1.87)	0.15	1.37 (0.96 – 1.99)	0.09
No	102	70 (69%)	32 (31%)		1		1	
**Infliximab (i.e., Remicade, Remsima, Flixabi, Inflectra, Zessly)**				0.29				
Yes	165	100 (61%)	65 (39%)		1.23 (0.87 - 1.75)	0.24	1.25 (0.87 - 1.79)	0.22
No	336	220 (65%)	116 (35%)		1		1	
**High-risk comorbidity**				0.34				
No high-risk comorbidity	289	188 (65%)	101 (35%)		1		1	
One high-risk comorbidity	132	88 (67%)	44 (33%)		0.93 (0.65 - 1.32)	0.69	1.10 (0.76 - 1.59)	0.61
Two high-risk comorbidities	45	25 (56%)	20 (44%)		1.49 (0.98 - 2.26)	0.06	2.21 (1.39 - 3.53)	**0.0008**
≥3 high-risk comorbidities	35	19 (54%)	16 (46%)		1.57 (1.04 - 2.37)	**0.03**	2.84 (1.73 - 4.70)	**<0.0001**
**SIRSCO[d] index for severe COVID-19**				0.59				
0= no increased risk	58	40 (69%)	18 (31%)		0.83 (0.45 – 1.48)	0.54	0.72 (0.39 – 1.29)	0.28
1= mild risk	248	161 (65%)	87 (35%)		1		1	
2= moderate risk	116	73 (63%)	43 (37%)		1.09 (0.82 – 1.45)	0.55	1.24 (0.92 – 1.68)	0.16
≥3= severe to very severe risk	79	46 (58%)	33 (42%)		1.33 (0.99 – 1.77)	0.06	2.23 (1.57 – 3.18)	**<0.0001**
**Sleep disturbances**				0.43				
Yes	112	68 (61%)	44 (39%)		1.19 (0.79 – 1.78)	0.40	1.17 (0.77 – 1.76)	0.46
No	386	250 (65%)	136 (35%)		1		1	

Factors, N (row %)	Total cohort, N=501	Low perceived risk (rating ≤ 3) n=320 (64%)	High perceived risk (rating > 3) n=181 (36%)	p-value[a]	Crude OR (95%CI)	p-value[b]	OR adjusted for age and gender (95%CI)	p-value[c]
**Weight change**				**0.02**				
Yes, intended	71	42 (59%)	29 (41%)		1.54 (0.93 – 2.51)	0.09	1.51 (0.90 – 2.50)	0.11
Yes, unintended	135	75 (56%)	60 (44%)		1.79 (1.35 – 2.36)	**<.0001**	1.82 (1.37 – 2.41)	**<0.0001**
Unchanged	294	203 (69%)	91 (31%)		1		1	
**Food intake**				**0.02**				
More	72	36 (50%)	36 (50%)		2.04 (1.25 – 3.32)	**0.004**	1.91 (1.17 – 3.13)	**0.009**
Unchanged	395	265 (67%)	130 (33%)		1		1	
Less	33	19 (58%)	14 (42%)		1.5 (0.98 – 2.29)	0.06	1.36 (0.88 – 2.09)	0.16
**COVID-19 PCR for patient**				0.62				
Yes	117	77 (66%)	40 (34%)		0.9 (0.59 – 1.34)	0.60	0.83 (0.54 – 1.26)	0.39
No	384	243 (63%)	141 (37%)		1		1	
**COVID-19 PCR for family member**				0.37				
Yes	99	67 (68%)	32 (32%)		0.81 (0.51 – 1.25)	0.34	0.74 (0.47 – 1.16)	0.20
No	398	250 (63%)	148 (37%)		1		1	
**Appointment declined by hospital**				0.17				
Yes, at least once	46	24 (52%)	22 (48%)		1.61 (0.88 – 2.95)	0.12	1.68 (0.91 – 3.10)	0.09
No, I could schedule all requested appointments	323	206 (64%)	117 (36%)		1		1	
I haven't asked for new appointment	130	88 (68%)	42 (32%)		0.84 (0.64 – 1.1)	0.20	0.83 (0.63 – 1.08)	0.16
**Appointment postponed**				**0.004**				
Yes	133	71 (53%)	62 (47%)		1.82 (1.25 – 2.65)	**0.002**	1.79 (1.22 – 2.62)	**0.003**
No (all reasons)	367	248 (68%)	119 (32%)		1		1	
**Interest in future telemedicine**				0.79				
Yes	247	157 (64%)	90 (36%)		1.05 (0.76 – 1.44)	0.76	1.05 (0.76 – 1.44)	0.78
No	252	163 (65%)	89 (35%)		1		1	
**Quality of life obtained in pediatric patients with Impact III Questionnaire, mean (SD), N=66**								
Impact III total score	76 (10)	77 (10)	73 (10)	0.09	0.97 (0.92 - 1.02)	0.19	0.97 (0.92 - 1.02)	0.26
Impact III well being	77 (14)	78 (14)	74 (13)	0.18	0.98 (0.94 - 1.02)	0.28	0.99 (0.95 - 1.02)	0.42
Impact III emotion	63 (18)	65 (18)	61 (16)	0.44	0.99 (0.96 - 1.02)	0.45	0.99 (0.95 - 1.02)	0.36
Impact III social	87 (11)	89 (10)	82 (11)	**0.02**	0.95 (0.90 - 0.99)	**0.03**	0.95 (0.9 – 1.00)	0.05
Impact III body image	71 (15)	69 (16)	75 (11)	0.20	1.03 (0.99 - 1.07)	0.15	1.03 (0.99 - 1.07)	0.12
**Quality of life obtained in patients aged ≥18 with SIBDQ, mean (SD), N=419**								
SIBDQ total score	53 (11)	55 (11)	52 (11)	**0.001**	0.98 (0.96 - 0.99)	**0.006**	0.98 (0.96 - 0.99)	**0.009**
Systemic Systems	5 (1)	5 (1)	5 (1)	**0.0008**	0.78 (0.67 - 0.90)	**0.001**	0.80 (0.68 - 0.92)	**0.003**
Social Function	6 (1)	6 (1)	6 (1)	0.6976	1.01 (0.87 - 1.18)	0.85	1.01 (0.86 - 1.18)	0.94
Bowel Systems	6 (1)	6 (1)	5 (1)	**0.0054**	0.84 (0.72 - 0.98)	**0.02**	0.82 (0.70 - 0.96)	**0.01**
Emotion Health	5 (1)	5 (1)	5 (1)	**0.0003**	0.77 (0.66 - 0.89)	**0.0005**	0.78 (0.67 - 0.91)	**0.002**
**Perceived Stress assessed by the mother of pediatric patients using PSQ, mean (95%CI), N=81**								
PSQ Total Score	42 (38 - 46)	41 (36 - 46)	43 (37 - 50)	0.60	1.006 (0.98 - 1.04)	0.64	1.01 (0.98 - 1.04)	0.47
PSQ Worries	30 (26 - 35)	30 (24 -36)	31 (26 - 37)	0.47	1.003 (0.98 - 1.03)	0.78	1.00 (0.98 - 1.03)	0.72
PSQ Tension	42 (37 - 47)	41 (36 - 47)	43 (34 - 53)	0.96	1.004 (0.98 - 1.03)	0.73	1.01 (0.98 - 1.03)	0.53
PSQ Joy	54 (50 - 59)	54 (49 - 60)	54 (45 - 63)	0.96	1.00 (0.98 - 1.02)	0.98	1.00 (0.97 - 1.02)	0.77
PSQ Demands	50 (45 - 54)	49 (43 -55)	52 (45 - 60)	0.49	1.008 (0.99 - 1.03)	0.50	1.01 (0.99 - 1.04)	0.30

Factors, N (row %)	Total cohort, N=501	Low perceived risk (rating ≤ 3) n=320 (64%)	High perceived risk (rating > 3) n=181 (36%)	p-value[a]	Crude OR (95%CI)	p-value[b]	OR adjusted for age and gender (95%CI)	p-value[c]
**Perceived Stress assessed by adult patients using PSQ, mean (95%CI), N=393**								
PSQ Total Score	37 (34 - 39)	33 (31 - 36)	42 (39 - 45)	**<0.0001**	1.02 (1.01 - 1.03)	**<0.0001**	1.02 (1.009 - 1.03)	**0.0003**
PSQ Worries	28 (26 - 30)	24 (22 - 27)	33 (29 - 37)	**0.0004**	1.02 (1.01 - 1.03)	**0.0001**	1.02 (1.007 - 1.03)	**0.0007**
PSQ Tension	40 (37 - 42)	36 (33 - 40)	46 (42 - 50)	**0.0003**	1.02 (1.01 - 1.02)	**0.0003**	1.01 (1.005 - 1.02)	**0.001**
PSQ Joy	59 (56 - 61)	61 (58 - 64)	54 (50 - 58)	**0.007**	0.989 (0.981 – 0.997)	**0.009**	0.989 (0.981 –0.997)	**0.01**
PSQ Demands	38 (36 - 40)	35 (32 - 38)	43 (40 - 47)	**0.0002**	1.02 (1.01 - 1.02)	**0.0007**	1.01 (1.00 - 1.02)	**0.004**

Results were presented in frequency (n) and row percentage (%) for categorial variables, mean with 95% confidence intervals (95% CI), mean with standard deviation (SD) or median with interquartile range (IQR) for continuous variables as appropriate.
Crude odds ratios (OR) with 95% CI obtained from the univariate logistic regression model are given.
Adjusted OR resulted from the same logistic regression model as the univariate analysis adjusted for age and gender.
[a] P-values refer to the comparison between group with low perceived risk (rating ≤ 3) versus group with high perceived risk (rating >3) obtained by Mann-Whitney-U-test for continuous variables, Pearson's Chi-square test, or Fisher's exact test for categorial variables, where applicable.
[b] P-values calculated from the Wald Chi-Square Test for the significance of crude OR.
[c] P-values calculated from the Wald Chi-Square Test for the significance of adjusted OR.
[d] SIRSCO (Scoring for increased risk for severe COVID-19 outcome) was described in Methods 2.3 and S2.
PSQ, Perceived stress questionnaire; SIBDQ, Short Inflammatory Bowel Disease Questionnaire
Bold p-values indicate statistically significant results with a p-value ≤ 0.05.

Supplementary Table S3: Univariate analysis of factors associated with high perceived harm in case of COVID-19 infection, N=501

Factors, N (row %)	Total cohort, N=501	Low perceived harm (rating ≤ 3) n=123 (25%)	High perceived harm (rating > 3) n=378 (75%)	p-value[a]	Crude OR (95%CI)	p-value[b]	OR adjusted for age and gender (95%CI)	p-value[c]
**Age in years, median (IQR)**	40 (27 – 54)	34 (22-48)	42 (29 – 55)	**0.0006**	1.02 (1.01 – 1.03)	**0.002**		
**Age groups**				**0.02**				
6 – 20	90	27 (30%)	63 (70%)		0.57 (0.34 – 0.99)	**0.04**		
>20 – 40	167	51 (31%)	116 (69%)		0.56 (0.38 – 0.81)	**0.002**		
>40 – 60	173	31 (18%)	142 (82%)		1.13 (0.78 – 1.62)	0.53		
>60	71	14 (20%)	57 (80%)		1			
**Gender**				0.72				
Female	231	55 (24%)	176 (76%)		1			
Male	270	68 (25%)	202 (75%)		0.93 (0.64 – 1.33)	0.69		
**Living in Munich**				0.62				
Yes	174	45 (26%)	129 (74%)		1		1	
No	327	78 (24%)	249 (76%)		1.11 (0.75 – 1.63)	0.58	1.13 (0.76 – 1.66)	0.52
**Housing space per person**				0.73				
≤30m2 – small space	140	31 (22%)	109 (78%)		1		1	
30m2 to 55m2 – medium space	239	59 (25%)	180 (75%)		0.87 (0.55 – 1.35)	0.54	0.76 (0.48 – 1.19)	0.24
>55m2 – large space	118	31 (26%)	87 (74%)		0.8 (0.5 – 1.26)	0.34	0.61 (0.37 – 0.98)	**0.047**
**Number of household members**				0.40				
1 to 2	254	58 (23%)	196 (77%)		1		1	
≥3	245	64 (26%)	181 (74%)		0.84 (0.58 – 1.19)	0.33	1.15 (0.78 – 1.7)	0.47
**Employment status**				0.71				
Employed	314	74 (24%)	240 (76%)		0.87 (0.59 – 1.27)	0.46	1.2 (0.78 – 1.83)	0.41
Self-employed	31	8 (26%)	23 (74%)		0.77 (0.41 – 1.48)	0.41	1.03 (0.54 – 2.03)	0.94
Not working	95	20 (21%)	75 (79%)		1		1	
**Net family income**				0.2				
≤2500 €	105	27 (26%)	78 (74%)		1.31 (0.8 – 2.18)	0.29	1.03 (0.62 – 1.75)	0.91
2500 to 4000 €	109	22 (20%)	87 (80%)		1.79 (1.2 – 2.71)	**0.005**	1.53 (1.01 – 2.34)	**0.046**
4000 to 6000 €	114	22 (19%)	92 (81%)		1.89 (1.32 – 2.73)	**0.0005**	1.88 (1.31 – 2.72)	**0.001**
>6000 €	77	24 (31%)	53 (69%)		1		1	
**Level of education**				0.74				
<12 years education	201	50 (25%)	151 (75%)		1		1	
≥12 years education	283	69 (24%)	214 (76%)		1.03 (0.7 – 1.48)	0.89	1.28 (0.86 – 1.89)	0.22
**Risk employment for COVID-19 infection**				0.78				
Yes	209	50 (24%)	159 (76%)		1.06 (0.74 – 1.54)	0.76	1.17 (0.81 – 1.71)	0.42
No	292	73 (25%)	219 (75%)		1		1	
**Public transport usage reduced**				**0.03**				
No	**59**	**22 (37%)**	**37 (63%)**		1		1	
Yes	**291**	**62 (21%)**	**229 (79%)**		2.2 (1.23 – 3.83)	**0.006**	2.53 (1.4 – 4.48)	**0.002**
Does not concern me	**148**	**38 (26%)**	**110 (74%)**		1.72 (0.96 – 3.02)	0.06	1.84 (1.02 – 3.28)	**0.04**

Factors, N (row %)	Total cohort, N=501	Low perceived harm (rating ≤ 3) n=123 (25%)	High perceived harm (rating > 3) n=378 (75%)	p-value[a]	Crude OR (95%CI)	p-value[b]	OR adjusted for age and gender (95%CI)	p-value[c]
**Temporary incapacity to work since 03/20**				0.80				
No	304	72 (24%)	232 (76%)		1		1	
Yes, due to IBD or other reasons	111	25 (23%)	86 (77%)		1.07 (0.67 – 1.76)	0.79	1.12 (0.7 – 1.86)	0.65
**BMI**				0.27				
Underweight	40	11 (28%)	29 (73%)		0.97 (0.49 – 2.08)	0.94	1.24 (0.61 – 2.68)	0.57
Normal weight	275	74 (27%)	201 (73%)		1		1	
Overweight	128	28 (22%)	100 (78%)		1.32 (0.97 – 1.79)	0.08	1.15 (0.83 – 1.59)	0.40
Obesity	57	9 (16%)	48 (84%)		1.96 (1.33 – 2.97)	**0.001**	1.58 (1.05 – 2.43)	**0.03**
**IBD Type**				0.32				
Crohn's Disease	295	66 (22%)	229 (78%)		1.24 (0.87 – 1.77)	0.24	1.17 (0.82 – 1.68)	0.39
Ulcerative Colitis / IBD-u	198	52 (26%)	146 (74%)		1		1	
**Disease activity (self-assessment)**				0.11				
Remission	228	65 (29%)	163 (71%)		1		1	
Mild activity	197	39 (20%)	158 (80%)		1.62 (1.1 – 2.36)	**0.01**	1.57 (1.07 – 2.3)	**0.02**
Moderate / severe activity	75	18 (24%)	57 (76%)		1.26 (0.83 – 1.93)	0.28	1.14 (0.75 – 1.76)	0.54
**Duration of IBD (to 2020)**				0.30				
0 to 5 years	126	35 (28%)	91 (72%)		1		1	
6 to 10 years	93	23 (25%)	70 (75%)		1.17 (0.7 – 1.95)	0.55	1.07 (0.63 – 1.79)	0.81
11 to 20 years	147	38 (26%)	109 (74%)		1.1 (0.7 – 1.71)	0.66	0.75 (0.46 – 1.2)	0.23
over 20 years	122	22 (18%)	100 (82%)		1.75 (1.1 – 2.73)	**0.02**	0.84 (0.48 – 1.46)	0.54
**Influenza vaccination**				0.16				
Yes	203	41 (20%)	162 (80%)		1.51 (1.03 – 2.25)	**0.04**	1.39 (0.95 – 2.08)	0.10
No	293	81 (28%)	212 (72%)		1		1	
**Any immunosuppressive medications used incl. IMM, biologics, steroids**				**<.001**				
Yes	399	84 (21%)	315 (79%)		2.32 (1.6 – 3.37)	**<0.0001**	2.44 (1.67 – 3.58)	**<0.0001**
No	102	39 (38%)	63 (62%)		1		1	
**Infliximab (i.e., Remicade, Remsima, Flixabi, Inflectra, Zessly)**				0.91				
Yes	165	41 (25%)	124 (75%)		0.98 (0.66 – 1.46)	0.91	1.1 (0.74 – 1.65)	0.66
No	336	82 (24%)	254 (76%)		1		1	
**High-risk comorbidity**				0.08				
No high-risk comorbidity	289	82 (28%)	207 (72%)		1		1	
One high risk comorbidity	132	28 (21%)	104 (79%)		1.47 (1 – 2.18)	**0.05**	1.31 (0.88 – 1.97)	0.19
Two high risk comorbidities	45	9 (20%)	36 (80%)		1.59 (0.98 – 2.63)	0.07	1.29 (0.76 – 2.22)	0.35
≥3 high risk comorbidities	35	4 (11%)	31 (89%)		3.07 (1.76 – 5.66)	**0.0002**	2.27 (1.21 – 4.44)	**0.01**
**SIRSCO[d] index for severe COVID-19 course**				**0.0006**				
0= no increased risk	58	21 (36%)	37 (64%)		0.74 (0.42 – 1.32)	0.29	0.72 (0.41 – 1.30)	0.26
1= mild	248	73 (29%)	175 (71%)		1			
2= moderate	116	17 (15%)	99 (85%)		2.43 (1.72 – 3.49)	**<0.0001**	2.28 (1.59 – 3.3)	**<0.0001**
≥3 severe to very severe risk	79	12 (15%)	67 (85%)		2.33 (1.63 – 3.37)	**<0.0001**	2.02 (1.33 – 3.09)	**0.001**
**Sleep disturbances**				**0.01**				
Yes	112	16 (14%)	96 (86%)		2.24 (1.33 – 4.03)	**0.004**	2.16 (1.27 – 3.89)	**0.01**
No	386	105 (27%)	281 (73%)		1		1	

Factors, N (row %)	Total cohort, N=501	Low perceived harm (rating ≤ 3) n=123 (25%)	High perceived harm (rating > 3) n=378 (75%)	p-value[a]	Crude OR (95%CI)	p-value[b]	OR adjusted for age and gender (95%CI)	p-value[c]
**Weight change**				**0.007**				
Yes, intended	71	15 (21%)	56 (79%)		1.54 (0.88 – 2.87)	0.15	1.92 (1.08 – 3.62)	**0.03**
Yes, unintended	135	21 (16%)	114 (84%)		2.24 (1.58 – 3.25)	**<.0001**	2.15 (1.51 – 3.13)	**<0.0001**
Unchanged	294	86 (29%)	208 (71%)		1		1	
**Food intake since pandemic**				0.15				
More	72	11 (15%)	61 (85%)		1.93 (1.04 – 3.94)	0.05	2.01 (1.07 – 4.13)	0.04
Unchanged	395	102 (26%)	293 (74%)		1		1	
Less	33	9 (27%)	24 (73%)		0.93 (0.59 – 1.51)	0.76	0.96 (0.6 – 1.57)	0.87
**COVID-19 PCR for patient since 03/20**				0.86				
Yes	117	28 (24%)	89 (76%)		1.04 (0.67 – 1.67)	0.85	1.17 (0.75 – 1.89)	0.51
No	384	95 (25%)	289 (75%)		1		1	
**COVID-19 PCR for family member since 03/20**				0.06				
Yes	99	23 (23%)	76 (77%)		1.07 (0.66 – 1.78)	0.80	1.19 (0.73 – 2)	0.51
No	398	97 (24%)	301 (76%)		1		1	
**Appointment declined by hospital**				0.11				
Yes, at least once	46	8 (17%)	38 (83%)		1.41 (0.68 – 3.32)	0.39	1.31 (0.62 – 3.09)	0.51
No, I could schedule all requested appointments	323	74 (23%)	249 (77%)		1		1	
I haven't asked for new appointment	130	40 (31%)	90 (69%)		0.67 (0.5 – 0.89)	**0.005**	0.67 (0.5 – 0.89)	**0.006**
**Appointment postponed**				**0.001**				
Yes	133	19 (14%)	114 (86%)		2.37 (1.46 – 4.07)	**0.0009**	2.29 (1.4 – 3.94)	**0.002**
No (all reasons)	367	104 (28%)	263 (72%)		1		1	
**Interest in future telemedicine**				0.55				
Yes	247	58 (23%)	189 (77%)		1.13 (0.8 – 1.62)	0.49	1.18 (0.83 – 1.71)	0.36
No	252	65 (26%)	187 (74%)		1		1	
**Quality of life obtained in pediatric patients with Impact III Questionnaire, mean (SD), N=66**								
Impact III total score	77 (14)	76 (15)	77 (14)	0.97	1.004 (0.95 – 1.06)	0.88	1.01 (0.96 – 1.07)	0.64
Impact III well being	63 (18)	63 (21)	64 (16)	0.91	1.004 (0.97 – 1.04)	0.81	1.02 (0.97 – 1.06)	0.45
Impact III emotion	87 (11)	88 (12)	86 (11)	0.98	1.003 (0.97 – 1.03)	0.85	0.998 (0.97 – 1.03)	0.92
Impact III social	71 (15)	68 (16)	72 (14)	0.37	0.98 (0.93 – 1.03)	0.45	0.99 (0.94 – 1.04)	0.63
Impact III body image	76 (10)	76 (11)	76 (10)	0.22	1.02 (0.99 – 1.06)	0.23	1.03 (0.99 – 1.07)	0.14
**Quality of life obtained in patients aged ≥18 with SIBDQ, mean (SD), N=419**								
SIBDQ total score	53 (11)	56 (11)	53 (11)	**0.0008**	0.97 (0.95 – 0.99)	**0.005**	0.97 (0.95 – 0.99)	**0.01**
Systemic Systems	5 (1)	6 (1)	5 (1)	**0.0006**	0.74 (0.61 – 0.88)	**0.001**	0.74 (0.61 – 0.89)	**0.001**
Social Function	6 (1)	6 (1)	6 (1)	**0.04**	0.87 (0.71 – 1.04)	0.15	0.89 (0.73 – 1.07)	0.24
Bowel Systems	6 (1)	6 (1)	6 (1)	**0.05**	0.86 (0.71 – 1.03)	0.10	0.88 (0.73 – 1.06)	0.18
Emotion Health	5 (1)	5 (1)	5 (1)	**0.001**	0.74 (0.61 – 0.89)	**0.001**	0.73 (0.6 – 0.88)	**0.001**
**Perceived Stress assessed by the mother of pediatric patients using PSQ, mean (95%CI), N=81**								
PSQ Total Score	42 (38 – 46)	40 (33 – 47)	43 (38 – 47)	0.74	1.01 (0.98 – 1.04)	0.58	1.01 (0.98 – 1.04)	0.44
PSQ Worries	30 (26 – 35)	28 (20 – 36)	31 (26 – 37)	0.55	1.01 (0.98 – 1.04)	0.50	1.01 (0.98 – 1.04)	0.60
PSQ Tension	42 (37 – 47)	39 (30 – 47)	43 (37 – 49)	0.49	1.01 (0.99 – 1.03)	0.40	1.01 (0.99 – 1.04)	0.38

Factors, N (row %)	Total cohort, N=501	Low perceived harm (rating ≤ 3) n=123 (25%)	High perceived harm (rating > 3) n=378 (75%)	p-value[a]	Crude OR (95%CI)	p-value[b]	OR adjusted for age and gender (95%CI)	p-value[c]
PSQ Joy	54 (50 – 59)	56 (48 – 63)	54 (48 – 60)	0.76	0.995 (0.973 – 1.017)	0.66	0.99 (0.97 – 1.01)	0.44
PSQ Demands	50 (45 – 54)	49 (38 – 59)	51 (46 – 56)	0.77	1.01 (0.98 – 1.03)	0.70	1.01 (0.99 – 1.04)	0.40
**Perceived Stress assessed by adult patients using PSQ, mean (95%CI), N=393**								
PSQ Total Score	37 (34 – 39)	30 (26 – 34)	39 (36 – 41)	**0.0007**	1.02 (1.01 – 1.04)	**0.0004**	1.03 (1.01 – 1.04)	**0.0001**
PSQ Worries	28 (26 – 30)	20 (17 – 24)	30 (28 – 33)	**<.0001**	1.03 (1.01 – 1.04)	**<.0001**	1.03 (1.02 – 1.04)	**<0.0001**
PSQ Tension	40 (37 – 42)	35 (30 – 39)	42 (39 – 45)	**0.03**	1.011 (1.002 – 1.02)	**0.02**	1.013 (1.003 – 1.023)	**0.01**
PSQ Joy	59 (56 – 61)	67 (62 – 72)	56 (53 – 59)	**0.0002**	0.98 (0.97 – 0.99)	**0.0003**	0.98 (0.97 – 0.99)	**0.0003**
PSQ Demands	38 (36 – 40)	33 (29 – 38)	40 (37 – 42)	**0.02**	1.012 (1.002 – 1.02)	**0.02**	1.015 (1.004 – 1.026)	**0.007**

Results were presented in frequency (n) and row percentage (%) for categorial variables, mean with 95% confidence intervals (95% CI), mean with standard deviation (SD) or median with interquartile range (IQR) for continuous variables as appropriate.
Crude odds ratios (OR) with 95% CI obtained from the univariate logistic regression model are given.
Adjusted OR resulted from the same logistic regression model as the univariate analysis adjusted for age (years) and gender.
[a] P-values refer to the comparison between group with low perceived harm (rating ≤ 3) versus group with high perceived harm (rating >3) obtained by Mann-Whitney-U-test for continuous variables, Pearson's Chi-square test, or Fisher's exact test for categorial variables, where applicable.
[b] P-values calculated from the Wald Chi-Square Test for the significance of crude OR.
[c] P-values calculated from the Wald Chi-Square Test for the significance of adjusted OR.
[d] SIRSCO (Scoring for increased risk for severe COVID-19 outcome) was described in Methods 2.3 and S2.
PSQ, Perceived stress questionnaire; SIBDQ, Short Inflammatory Bowel Disease Questionnaire
Bold p-values indicate statistically significant results with a p-value ≤ 0.05.

Supplementary Table S4: Considered variables for multivariable logistic regression

A univariate logistic analysis was performed including all subjects to determine the associations between variables of interest and high perceived risk or high perceived harm. All statistically significant variables associated with high perceived risk or high perceived harm ($p \le 0.25$) in the univariate analysis were considered in the multivariable logistic regression. Reference category (ref.) was chosen according to clinician interest or as the most frequent category present in the considered variables.

Domains	Variable of interest
Demographics	Age in years
	Gender, categorized in Male vs. Female (ref.)
	Living in Munich, categorized in Yes (ref.) vs. No
Socio-economics	Housing space per person, categorized in<br>• small space (≤30m^2 per person) (ref.)<br>• middle space (30m^2 to 55m^2)<br>• large space (>55m^2)
	Number of family members per household, categorized in<br>• 1-2 persons per household (ref.)<br>• ≥3 persons per household
	Current employment status, categorized in<br>• Employed<br>• Self-employed<br>• Not working includes unemployed, retired, parental leave, sabbatical, students (ref.)
	Level of education according to the German school system, categorized in<br>• <12 years education (ref.)<br>• ≥12 years education
COVID-19 related factors	High-risk job for COVID-19 infection was considered, if patients answered as employees in the healthcare sector (e.g., nursing home, hospital, general praxis), emergency service (e.g., fire department, police), airport and air traffic (pilots, flight attendants), public transport (e.g., bus driver, train attendant, subway driver), education (e.g., kindergarten, day care, school), sales (e.g., grocery store, hardware store, drugstore), social work and other employment involved contacts with people outside of their own families.<br>Answers: Yes vs. No (ref.)
	Public transport usage reduced<br>• No (ref.)<br>• Yes<br>• No need
	COVID-19 PCR for patient since 03/20: Yes vs. No (ref.)
	COVID-19 PCR for family member since 03/20: Yes vs. No (ref.)
Health conditions	BMI (kg/m^2) was calculated for all adult patients ≥20 years, categorized in 4 nutrition status according to WHO.<br>• Underweight for BMI < 18.5 kg/m^2<br>• Normal weight for BMI in 18.5–24.9 kg/m^2 (ref.)<br>• Overweight for BMI in 25.0–29.9 kg/m^2<br>• Obesity for BMI ≥ 30 kg/m^2<br>BMI Z-scores for all children aged ≥6 to <20 was calculated based on WHO/CDC Growth chart 2000. Interpretation of cut-offs as follows<br>• Underweight/Wasting <-1SD<br>• Normal weight: ≥-1SD and <+1SD<br>• Overweight: ≥+1SD<br>• Obesity: ≥+2SD
	IBD Type: Crohn's Disease and Ulcerative Colitis / IBD-u (ref.)
	Disease activity (self-assessment)<br>• Remission (ref.)<br>• Mild activity<br>• Moderate / severe activity
	Duration of IBD (to 2020)<br>• 0 to 5 years (ref.)<br>• 6 to 10 years<br>• 11 to 20 years<br>• over 20 years

Domains	Variable of interest
	Influenza vaccination: Yes vs. No (ref.)
	Any immunosuppressive medications used incl. IM, biologics, steroids: Yes vs. No (ref.)
	Infliximab (i.e., Remicade, Remsima, Flixabi, Inflectra, Zessly): Yes vs. No (ref.)
	High-risk comorbidity (S2)<br>• No high-risk comorbidity (ref.)<br>• One high risk comorbidity<br>• Two high risk comorbidities<br>• ≥3 high risk comorbidities
	SIRSCO index for severe COVID-19 (S2)<br>• 0= no increased risk<br>• 1= mild risk (ref.)<br>• 2= moderate risk<br>• ≥3= severe to very severe risk
Lifestyle factors	Sleep disturbances since pandemic: Yes vs. No (ref.)
	Weight change, independent if weight gain or weight loss.<br>• Yes, intended<br>• Yes, unintended<br>• Unchanged (ref.)
Clinical care	Appointment declined by hospital<br>• Yes, at least once<br>• No, I could schedule all requested appointments (ref.)<br>• I haven't asked for new appointment
	Appointment postponed: Yes vs. No with all reasons (ref.)
	Interest in future telemedicine: Yes vs. No (ref.)

Employment status correlates significantly with net income per household ($p<0.0001$) and temporary incapacity to work since 3/20 ($p= 0.004$). Due to missing data and to avoid multicollinearity, net income per household and temporary incapacity to work since 3/20 were excluded in the stepwise selection for multivariable logistic regression.

Food intake and weight change were highly significant correlated ($p <.0001$). Food intake was excluded in the stepwise selection for multivariable logistic regression due to its possibly greater recall bias than weight change.
